# Progression of basal ganglia pathology in heterozygous Q175 knock‐in Huntington's disease mice

**DOI:** 10.1002/cne.25023

**Published:** 2020-09-20

**Authors:** Yunping Deng, Hongbing Wang, Marion Joni, Radhika Sekhri, Anton Reiner

**Affiliations:** ^1^ Department of Anatomy and Neurobiology The University of Tennessee Health Science Center Memphis Tennessee USA; ^2^ Department of Pathology The University of Tennessee Health Science Center Memphis Tennessee USA; ^3^ Department of Ophthalmology The University of Tennessee Health Science Center Memphis Tennessee USA

**Keywords:** aggregates, basal ganglia, Huntington's disease, neuropathology, Q175 mice

## Abstract

We used behavioral testing and morphological methods to detail the progression of basal ganglia neuron type‐specific pathology and the deficits stemming from them in male heterozygous Q175 mice, compared to age‐matched WT males. A rotarod deficit was not present in Q175 mice until 18 months, but increased open field turn rate (reflecting hyperkinesia) and open field anxiety were evident at 6 months. No loss of striatal neurons was seen out to 18 months, but ENK+ and DARPP32+ striatal perikarya were fewer by 6 months, due to diminished expression, with further decline by 18 months. No reduction in SP+ striatal perikarya or striatal interneurons was seen in Q175 mice at 18 months, but cholinergic interneurons showed dendrite attenuation by 6 months. Despite reduced ENK expression in indirect pathway striatal perikarya, ENK‐immunostained terminals in globus pallidus externus (GPe) were more abundant at 6 months and remained so out to 18 months. Similarly, SP‐immunostained terminals from striatal direct pathway neurons were more abundant in globus pallidus internus and substantia nigra at 6 months and remained so at 18 months. FoxP2+ arkypallidal GPe neurons and subthalamic nucleus neurons were lost by 18 months but not prototypical PARV+ GPe neurons or dopaminergic nigral neurons. Our results show that striatal projection neuron abnormalities and behavioral abnormalities reflecting them develop between 2 and 6 months of age in Q175 male heterozygotes, indicating early effects of the HD mutation. The striatal pathologies resemble those in human HD, but are less severe at 18 months than even in premanifest HD.

## Graphical abstract

Striatal cholinergic (ChAT+) interneurons in WT and heterozygous Q175 mice at 18 months of age. The images show that striatal cholinergic interneuron abundance is comparable, but dendrites in Q175 mice are significantly fewer and shorter. These abnormalities and diverse striatal projection neuron abnormalities develop between 2 and 6 months of age in Q175 heterozygotes, indicating early effects of the HD mutation on the striatum in these mice that in many regards resemble those in human HD. Nonetheless, the pathologies do not yet involve overt striatal neuron loss even by 18 months, indicating they are less severe than even in premanifest HD.

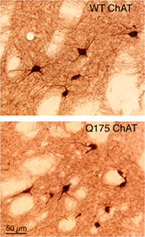

## 1 INTRODUCTION

Progressive degeneration of specific neuron types within the striatal and pallidal parts of the basal ganglia is a well‐known feature of Huntington's disease (HD; Deng et al., 2004; Reiner, Dragatsis, & Dietrich, 2011). Among the striatal events in adult‐onset HD is the earlier loss of enkephalinergic indirect pathway striatal projection neurons (iSPNs) than substance P‐containing direct pathway striatal projection neurons (dSPNs). In particular, the iSPNs projecting to the external pallidal segment (GPe) are lost earlier in HD progression than are the dSPNs projecting to the internal pallidal segment (GPi; Deng et al., 2004; Reiner & Deng, 2018). The dSPNs projecting to the substantia nigra pars reticulata (SNr), however, appear as vulnerable as the iSPNs, and are lost at a comparable rate as the iSPNs. Pallidal neurons in human HD show upregulation of GABAA receptors in response to loss of inhibitory input as striatal projection neurons die, with the pattern of upregulation in globus pallidus (i.e., GPe vs GPi) reflecting the pattern of striatal projection neuron loss—that is upregulation in GPe occurs at earlier grades than upregulation in GPi due to the greater loss of iSPNs projecting to GPe than dSPNs projecting to GPi (Glass, Dragunow, & Faull, 2000; Reiner ‐ Dragatsis, & Dietrich, 2011). Among striatal interneurons, the tonically active large cholinergic interneurons (TANs) and the low threshold spiking (LTS) interneurons co‐containing somatostatin (SS), neuropeptide Y (NPY), and neuronal nitric oxide synthase (nNOS) survive the HD disease process, while the fast‐spiking GABAergic interneurons (FSIs) containing parvalbumin (PARV+) are largely as vulnerable as iSPNs (Reiner, Dragatsis, & Dietrich, 2011; Reiner et al., 2013).

A variety of transgenic and knock‐in models have been developed to better understand HD pathogenesis, pathophysiology, and treatment choices, with knock‐in mice that express mutant huntingtin from the endogenous mouse site regarded as providing the closest genetic mimic of HD (Heng, Tallaksen‐Greene, et al., 2008; Menalled et al., 2014). Among these, the Q175 mouse has emerged as particularly important model for testing of therapeutics, but the extent to which their basal ganglia pathology matches that in human adult‐onset HD is not known. The Q175 mice represent a line derived from the Zeitlin knock‐in Q140 mice, with a germline expansion of the CAG tract (Heikkinen et al., 2012; Menalled et al., 2012). Both the original Zeitlin mice and the Q175 mice encode the human exon 1 sequence in the context of a full‐length mouse *Htt* locus. Subsequent to its derivation as a line with 175 CAG repeats, the CAG repeat number in the Q175 mice as maintained at JAX (Bar Harbor, ME) expanded further, and a line was selectively stabilized at a CAG repeat of approximately 190. This line, nonetheless, is still referred to as the Q175. Although some data are available on the progression of striatal and subthalamic nucleus pathology in the Q175 mouse (Atherton et al., 2016; Bertoglio et al., 2018; Heikkinen et al., 2012; Rothe et al., 2015; Smith et al., 2014), no specific information is available on the progression of type‐specific SPN and striatal interneuron pathology. To this end, we used immunolabeling of striatal terminals in striatal target areas (for SP, ENK, or DARPP32), immunolabeling of cholinergic, nNOS+, and PARV+ striatal interneurons, in situ hybridization histochemistry (ISHH) for dSPNs (substance P) and iSPNs (enkephalin), ISHH and immunolabeling for GABAA receptors in GPe, GPi, and SNr, immunostaining of striatal projection neuron perikarya for DARPP32, and staining for pan‐neuronal markers (such as by NeuN immunolabeling or by cresyl violet staining), and stereological and morphometric methods to detail the progression of striatal neuron type‐specific pathology and loss in 2‐, 6‐, 12‐, and 18‐month‐old heterozygous Q175 males. Because neurochemical pathology and neuron loss in globus pallidus, substantia nigra and subthalamic nucleus (STN) occur and may also contribute to HD symptoms (Reiner, Dragatsis, & Dietrich, 2011; Vonsattel, 2008), we also characterized their pathology in the Q175 mice, using ISHH and immunolabeling for parvalbumin (PARV) in GPe, GPi and SNr, FoxP2 immunolabeling of GPe arkypallidal neurons, tyrosine hydroxylase immunolabeling of SNc dopaminergic neurons, and cresyl violet staining of STN neurons. We also quantified aggregate load in striatum and cerebral cortex as part of our immunohistochemical assessment. To relate neuropathology to pathophysiology and pathogenesis, we assessed rotarod and open field performance prior to sacrificing the mice for neurochemical and morphological evaluation.

## 2 MATERIALS AND METHODS

Heterozygous male Q175 HD mice were obtained from JAX at approximately 2 months of age (30 WT, 30 Q175), 6 months of age (30 WT, 30 Q175), 12 months of age (30 WT, 29 Q175), and 18 months of age (27 WT, 25 Q175; Bar Harbor, ME). The CAG repeat length at the mutant huntingtin site was assessed and found to be generally around 190 (Table 1). By convention, these HD mutant mice are nonetheless referred to as Q175 mice (Menalled et al., 2014). The genotype analysis was carried out by the Laragen Corporation (Culver City, CA). The cohorts were sacrificed on average at 1.9 months, 6.3 months of age, 13.3 months of age, and 18.1 months of age. For convenience, these are referred to as 2‐, 6‐, 12‐, and 18‐month‐old animals. All animal use was carried out in accordance with the National Institutes of Health Guide for Care and Use of Laboratory Animals, Society for Neuroscience Guidelines, and the University of Tennessee Health Science Center Guidelines. We focused on males since prior studies had found no major differences between males and females in the Q175 phenotype (Goodliffe et al., 2018; Menalled et al., 2012; Padovan‐Neto et al., 2019; Rothe et al., 2015), and because our funding source accordingly had requested we focus our studies on heterozygous males.

**TABLE 1:** Mice numbers, sacrifice age, weight at sacrifice, and CAG repeat length for WT and Q175 used in this study

Group	Number of mice	Mean sacrifice age in months	Mean body weight (g) at sacrifice	Mean CAG
WT	30	1.9	23.1 ± 0.26	NA
Q175	30	1.9	22.5 ± 0.19	192.5 ± 0.64
WT versus Q175 *t*‐test			0.071509	
WT	30	6.3	31.5 ± 0.29	NA
Q175	30	6.3	30.3 ± 0.51	192.1 ± 0.77
WT versus Q175 *t*‐test			0.058040	
WT	30	13.2	36.8 ± 0.95	NA
Q175	29	13.3	27.9 ± 0.45	192.1 ± 1.03
WT versus Q175 *t*‐test			1.985E‐11	
WT	27	18.1	31.9 ± 0.33	NA
Q175	25	18.1	26.0 ± 0.26	192.5 ± 0.85
WT versus Q175 *t*‐test			1.6 × 10^–19^	

*Note*: Weight and CAG length are presented as the mean ± *SEM*. Unpaired two‐tailed *t*‐tests were used to assess the statistical significance of the weight differences between WT and Q175 mice.

### 2.1 Behavioral studies

#### 2.1.1 Rotarod

Rotarod analysis was carried out using a San Diego Instruments rodent rotarod (San Diego, CA), as described previously (Guley et al., 2016; Reiner, Lafferty, Wang, Del Mar, & Deng, 2012). For rotarod, revolutions per minute (RPM) increased from 0 to 30 over a 4‐min period, and 30 RPM was then maintained for another 2 min. The first rotarod session was a three‐trial training session, followed the next day by a three‐trial test session. Time to fall was the measure. Our rotarod task has proven sensitive to motor deficits in R6/2 mice and in mice with focal cranial blast mild traumatic brain injury (Guley et al., 2016; Reiner, Lafferty, et al., 2012).

#### 2.1.2 Open field

We conducted open field tests as described previously (Reiner, Lafferty, et al., 2012) using a Noldus EthoVision video tracking system (Noldus Information Technology, The Netherlands), and the SEE analysis software of Drai and Golani (2001). The circular arena had a 200 cm diameter, with a nonporous gray floor and a 50 cm gray wall, which provided contrast for video tracking of the mice. SEE dichotomizes mouse movements into lingering episodes and progression segments, and it distinguishes movements or lingering near the wall from those in the arena center (center defined as >15 cm from wall). SEE provides 37 endpoints related to locomotion, anxiety, and navigation, and is robust in identifying differences among mouse strains (Drai, Benjamini, & Golani, 2000; Drai & Golani, 2001; Kafkafi et al., 2003; Kafkafi, Mayo, Drai, Golani, & Elmer, 2001; Lipkind et al., 2004), and in detecting abnormalities in mouse models of human disease or brain trauma (Guley et al., 2016; Reiner, Lafferty, et al., 2012). We report here on the results for those open field parameters that are independent of others, are typically examined in studies of HD mouse models, and show a clear mutant—WT difference, particularly across the different age groups examined. Noteworthy endpoints not showing significant differences are also reported. Mice were weighed and sacrificed for morphological and neurochemical studies following behavioral testing.

### 2.2 Neurochemical and morphological studies

Neurochemical or histological analysis was carried out on fixed or unfixed tissue to determine: (a) the neurochemical integrity of iSPN projection systems to GPe and the dSPN projections to GPi and SN; (b) the overall striatal neuron abundance; (c) the overall relative SPN abundance; (d) the abundance and morphology of neurochemically defined types of striatal interneurons; (e) the abundance of neurochemically defined types of neurons in GPe, GPi, SNr, and substantia nigra pars compacta (SNc); (f) the abundance of neurons in the subthalamic nucleus (STN); (g) the expression of GABAA‐α1 by GPe, GPi and SNr neurons; and (h) the size and abundance of mutant protein neuronal intranuclear inclusion (NII) in cortex and striatum. Further details on the different lines of studies to pursue these goals follow. Note that all measurements presented below were done blinded as to genotype, and all Q175 images shown were image processed for brightness, contrast, and sharpness with Photoshop or Word in the same manner as the corresponding WT images.

#### 2.2.1 Fixed tissue and immunohistochemical studies

Immunohistochemical analysis was carried out on fixed tissue to specifically determine: (a) the integrity of the enkephalinergic (ENK+) iSPN projections to GPe and the substance P‐containing (SP+) dSPN projections to GPi and SN; (b) the overall striatal neuron abundance based on stereological analysis of cresyl violet‐stained and NeuN‐immunostained material; (c) the overall striatal projection neuron (SPN) abundance and the integrity of striatal projection systems as determined using DARPP32 immunolabeling; (d) the abundance of the tonically active cholinergic striatal interneurons, of the fast spiking parvalbuminergic (PARV+) striatal interneurons, and of the low threshold spiking (LTS) neuronal nitric oxide synthase‐containing (nNOS+) striatal interneurons as determined by stereological analysis; (e) the dendritic branching of cholinergic striatal interneurons by Sholl analysis of immunostained material; (f) the abundance of PARV+ pallidal type GABAergic projections neurons in GPe (prototypical), GPi, and SNr as determined by counts in PARV immunolabeled material; (g) the abundance of FoxP2+ arkypallidal neurons in GPe; (h) the abundance of dopaminergic neurons in the substantia nigra pars compacta (SNc) as determined from tyrosine hydroxylase immunostained material; (i) the abundance of neurons in the subthalamic nucleus (STN) as determined by stereological analysis of cresyl violet stained tissue; (j) the expression of GABAA‐α1 by GPe, GPi and SNr neurons as determined from immunostained material; and (k) the abundance and size of neuronal intranuclear inclusion (NII) in cortex and striatum as determined from sections immunostained for ubiquitin or with EM48. For these studies, Q175 and WT mice were deeply anesthetized (avertin; 0.2 ml/g body weight), and perfused transcardially with 40 ml of 0.9% NaCl in 0.1 M sodium phosphate buffer at pH 7.4 (PB), followed by 200 ml of 4% paraformaldehyde, 0.1 M lysine‐0.1 M sodium periodate in 0.1 M sodium phosphate buffer (pH 7.4). The brains were removed and stored in a 20% sucrose/10% glycerol solution at 4°C until sectioned frozen on a sliding microtome in the transverse (i.e., coronal) plane at 35 μm. Each brain was collected as 12 separate series in 0.1 M PB 0.02% sodium azide, and stored until processed for immunohistochemistry. A one in six series from each mouse was mounted as sectioned, and stained for cresyl violet.

The peroxidase–antiperoxidase (PAP) procedure was used to visualize the above‐noted markers in Q175 and WT brains (Dragatsis et al., 2009; Meade et al., 2002; Reiner et al., 2007). To enhance immunolabeling and/or reduce nonspecific background, sections were pretreated with 1% sodium borohydride in 0.1 M PB for 30 min followed by incubation in 0.5% H_2_O_2_ solution in 0.1 M PB for 30 min, as previously (Deng & Reiner, 2016). A brief description of the species host, source, immunogen, and specificity of each primary antibody used to detect the above markers follows. For each, the RRID number is provided as well, which provides a resource for further detail on each primary antibody. Note that specificity of all antibodies used here was confirmed by the antibody developer (whether commercial or private), by us in prior studies, and/or by comparison to the labeling patterns expected as shown in published studies and/or to those shown in the Allen Mouse Brain Atlas (2004).

Enkephalin (ENK) immunolabeling was used to detect iSPN terminals in GPe, using a rabbit polyclonal antibody (1:5,000 dilution) directed against leucine‐enkephalin (ImmunoStar, Hudson, WI; Cat# 20066; RRID: AB_572247), whose specificity has been shown previously (Reiner, 1987; Reiner et al., 2007). Substance P (SP) immunolabeling was used to study SP+ striatal terminals in GPi and SNr, using a rabbit polyclonal antibody (1:5,000 dilution) directed against SP (ImmunoStar, Hudson, WI; Cat# 20064; RRID: AB_572266), whose specificity has been documented previously (Figueredo‐Cardenas, Anderson, Chen, Veeman, & Reiner, 1994; Ruscheweyh, Forsthuber, Schoffnegger, & Sandkühler, 2007). Immunolabeling for DARPP32 (1:10,000 dilution) was carried out using a mouse monoclonal antibody against DARPP32 generated using bovine DARPP32 as immunogen (generously provided by P. Greengard and H. Hemmings; RRID: AB_2314285), whose specificity has been previously documented (Ouimet, Miller, Hemmings Jr., Walaas, & Greengard, 1984). Immunolabeling for NeuN, now known to be Fox‐3 (Kim, Adelstein, & Kawamoto, 2009), was used to detect neuronal perikarya for counting (1:2,500 dilution), using a rabbit monoclonal anti‐NeuN generated using aa 1–100 of synthetic human NeuN (Abcam, Cambridge, UK: Cat# AB177487; RRID: AB_2532109). Cholinergic striatal interneurons were visualized by immunolabeling (1:100 dilution) for choline acetyltransferase (ChAT) using a goat polyclonal antibody raised against human placental ChAT (Millipore, Burlington, MA; Cat# AB144P; RRID: AB_90650; Kagi, Berchtold, & Heizmann, 1987), fast‐spiking striatal interneurons were visualized by immunolabeling for PARV (1:5,000 dilution) using a mouse monoclonal antibody raised against carp muscle PARV (Sigma‐Aldrich, St. Louis, MO; Cat# P3171; RRID: AB_2313693; Celio, 1990; Reiner et al., 2007), and LTS striatal interneurons were visualized by immunolabeling (1:800 dilution) for neuronal nitric oxide synthase (nNOS) using a rabbit polyclonal antibody raised against the C‐terminus of rat nNOS (Santa Cruz, Dallas, TX; Cat# SC648; RRID: AB_630935; Giove, Deshpande, & Eldred, 2009). PARV immunolabeling with the above mouse monoclonal antibody was also used to detect prototypical pallidal‐type neurons in GPe, GPi, and SNr. A rabbit polyclonal antibody against FoxP2 generated using the 700 amino acids at the C‐terminus of human FOXP2 (1:5,000 dilution; Abcam, Cambridge, UK: Cat# AB16046; RRID: AB_2107107) was used to detect arkypallidal neurons in GPe (Abdi et al., 2015). The specificity of this antibody has been demonstrated previously (Özdinler et al., 2011). Dopaminergic neurons of SNc were detected using a mouse monoclonal antibody (1:1,000 dilution) of demonstrated specificity (Reyes et al., 2012) against tyrosine hydroxylase (TH) raised against aa 40–152 at the N‐terminus of rodent and human TH (Sigma‐Aldrich, St. Louis, MO; Cat# T2928; RRID: AB_477569). Because loss or hypoactivity of the striatal input to its target areas is associated with an upregulation of GABAA receptors, especially the α1 subunit (Glass et al., 2000), we assessed localization of this receptor subunit in GPe, GPi, and SNr using immunolabeling (1:100 dilution) with a rabbit polyclonal antibody raised against the cytoplasmic loop of the alpha 1‐subunit of rat GABAA (PhosphoSolutions, Aurora, CO; Cat# 811‐GA1C; RRID: AB_2492099). Immunolabeling was used to visualize mutant huntingtin protein aggregated in ubiquitinated neuronal intranuclear inclusions (NIIs) in Q175 mice (Davies et al., 1997), using a mouse monoclonal antibody against ubiquitin generated using bovine ubiquitin (1:10,000 dilution; Millipore, Burlington, MA; Cat# MAB1510; RRID: AB_2180556) or the EM48 mouse monoclonal antibody raised against the N‐terminus (aa 1–256) of huntingtin without the polyQ tract (1:250 dilution; Millipore, Burlington, MA; Cat# AB5374; RRID: AB_10055116). The specificity of these two antisera was detailed by us previously (Reiner et al., 2007). We used antigen retrieval (Jiao et al., 1999) to enhance detection of mutant protein aggregates, which involves using a water bath to heat the tissue to 80°C for 30 min in a pH 9.0 10 mM sodium citrate or Tris‐EDTA (ethylenediamine tetra‐acetic acid) buffer. We have consistently found, as we did in this case with our comparisons of EM48 and anti‐ubiquitin, that while immunolabeling with EM48 detects mutant protein aggregates well, anti‐ubiquitin is even more effective—with the aggregates detected being larger and more numerous. In light of this, we performed our analysis of mutant protein aggregates on tissue immunolabeled with anti‐ubiquitin after antigen retrieval.

#### 2.2.2 Fresh frozen tissue and immunohistochemical studies

The GPe has been shown to contain a diversity of neuron types in rodents and primates, with the two main types being called prototypical neurons and arkypallidal neurons. Prototypical neurons are more laterally situated in GPe, are GABAergic, have high, regular basal firing rates, express PARV and Nkx2.1, represent the majority of GPe neurons (about 55%), and account for the canonical inhibitory projection of GPe to STN (Abdi et al., 2015; Hegeman, Hong, Hernández, & Chan, 2016; Hernández et al., 2015; Mallet et al., 2012; Mallet, Delgado, Chazalon, Miguelez, & Baufreton, 2019; Sato, Lavallée, Lévesque, & Parent, 2000). The arkypallidal GPe neurons are more medially situated, are also GABAergic, have low, irregular firing rates, express Npas1 and FoxP2 but not Nkx2.1 or PARV, represent about 25% of GPe neurons in mice, and project back to the striatum, where they end mainly on SPNs (Abdi et al., 2015; Hegeman et al., 2016; Hernández et al., 2015; Mallet et al., 2019). Published reports had claimed that Lhx6 is a selective marker of arkypallidal GPe neurons (Mastro, Bouchard, Holt, & Gittis, 2014), but this claim was based on an Lhx6 reporter line of mouse in which Lhx6 expression does not match native Lhx6 expression (Abdi et al., 2015), which tends to be more widespread in GPe and include PARV expressing neurons that project to STN. Rather, more recent immunolabeling and ISHH studies show that arkypallidal neurons can be identified in GPe by their expression of FoxP2 (Abdi et al., 2015; Hernández et al., 2015). Note that a less abundant population of prototypical neurons accounting for about 15% of all GPe neurons, expresses Npas1 and Nkx2.1 but not PARV or FoxP2 and accounts for GPe projections to FSI‐type striatal interneurons, cerebral cortex, GPi, and SNr (Gittis et al., 2014; Hegeman et al., 2016; Mallet et al., 2019). In the present studies, we evaluated the effect of the Q175 mutation on arkypallidal GPe neurons using immunolabeling for FoxP2. Because of limitations on the availability of fixed tissue, the rabbit polyclonal antibody against FoxP2 was also used to define arkypallidal neurons in GPe in the fresh frozen tissue otherwise used for ISHH. The sections were cut, collected, and stored until used as described below in the ISHH section. On‐the‐slide immunolabeling used the PAP method as described in our prior studies (Kimble, Fitzgerald, & Reiner, 2006).

#### 2.2.3 Fixed tissue and immunofluorescence studies

We used immunofluorescence double immunolabeling on fixed WT tissue sections through GPe to confirm that FoxP2 was restricted to neurons in GPe, using double labeling with a mouse monoclonal anti‐NeuN (1:2,500 dilution) and the rabbit anti‐FoxP2. The mouse monoclonal anti‐NeuN (Millipore, Burlington, MA; Cat# MAB377; RRID: AB_2298772) was generated using the first 97aa of N‐terminal murine NeuN and its specificity has been documented by us previously (Reiner, Yang, Cagle, & Honig, 2011). We also confirmed that FoxP2 did not occur in PARV+ prototypical GPe neurons, using immunofluorescence double labeling with the above‐noted mouse anti‐PARV and rabbit anti‐FoxP2. FoxP2 was visualized with a secondary antibody conjugated to Alexa‐594, while NeuN and PARV were visualized with a secondary antibody conjugated to Alexa‐488 (Invitrogen). A Zeiss 710 confocal laser‐scanning microscope (CLSM; Carl Zeiss AG, Oberkochen, Germany) was used to image brain tissue prepared by immunofluorescence.

#### 2.2.4 Fresh frozen tissue and ISHH methods

We processed brain tissue that had been fresh‐frozen for ENK, SP, PARV, or GABAA‐α1 mRNA detection by ISSH (Dragatsis et al., 2018; Reiner, Lafferty, et al., 2012; Sun, Del Mar, Meade, Goldowitz, & Reiner, 2002; Wang et al., 2006). The ENK and SP ISHH were used to assess iSPN and dSPNs, respectively, while PARV ISHH was used to assess prototypical neurons of GPe, and GABAergic pallidal type projection neurons of GPi and SNr. GABAA‐α1 ISHH was used to assess expression of this receptor type subunit in GPe, GPi, and SNr. ISHH was performed on 20 μm thick fresh frozen cryostat sections sectioned on a Hacker‐Bright cryostat through the striatum, GPe, GPi, and SN. The sections were collected onto pre‐cleaned Superfrost®/Plus microscope slides as sectioned, dried on a slide warmer, and stored at −80°C until used for ISHH. To process tissue for ISHH, the slides were removed from −80°C, and quickly thawed and dried using a hair dryer. After fixation with 2% paraformaldehyde in saline sodium citrate (2× SSC) for 5 min, the sections were acetylated with 0.25% acetic anhydride/0.1 M triethanolamine hydrochloride (pH 8.0) for 10 min, dehydrated through a graded ethanol series, and air‐dried. Digoxigenin‐UTP labeled cRNA probes (i.e., riboprobes) for preproenkephalin (PPE), preprotachykinin (PPT), PARV, and GABAA‐α1 were transcribed from plasmids with PPE cDNA (817 bp), PPT cDNA (900 bp), PARV cDNA (823 bp), and GABAA‐α1 cDNA (769 bp) inserts, generated by us using RT‐PCR. Primers used for PCR to generate the cDNA are listed in Table 2. The PPE (ENK) riboprobe was directed against nucleotides 312–1,128 (GenBank accession number NM_001002927), while the PPT (SP) riboprobe was directed against nucleotides 95–994 (GenBank accession number D17584). The PARV probe was directed against nucleotides 2–824 (GenBank accession number NM_013645.2), and the GABAA‐α1 probe was directed against nucleotides 1,200–1,968 (GenBank accession number NM_010250.5). The sections were incubated with digoxigenin (DIG)‐labeled riboprobe in hybridization buffer containing 50% formamide, 4× SSC, 1× Denhardt's solution, 200 μg/ml denatured salmon sperm DNA, 250 μg/ml yeast tRNA, 10% dextran sulfate, and 20 mM dithiothreitol (DTT) at 63°C overnight. After hybridization, the slices were washed at 58°C in 4× SSC, 50% formamide with 2× SSC (twice), and then 2× SSC, followed by treatment with RNase A (20 μg/ml) for 30 min at 37°C. Sections were then washed at 55°C in 1× SSC, 0.5× SSC, 0.25× SSC, ethanol dehydrated, and air‐dried. Digoxigenin labeling was detected using anti‐digoxigenin Fab fragments conjugated to alkaline phosphatase (AP), as visualized with nitroblue tetrazolium (NBT; Roche, Indianapolis, IN). Sections were coverslipped with a 1% gelatin‐based aqueous solution. The PPE and PPT ISHH labeling is referred to here as ENK and SP labeling for simplicity and for congruence with the immunolabeling.

**TABLE 2:** Information on the riboprobes used in the current study to detect PPE, PPT, PARV, and GABAA‐α1: The riboprobe length, the primers used to generate them, the nucleotide sequence they target, and GenBank accession number for their target

Target mRNA	Riboprobe length	PCR primers	Nucleotide target	GenBank mRNA accession #
PPE	817 bp	Sense: 5′‐TTCCTGAGGCTTTGCACC‐3′ Antisense: 5′‐TCACTGCTGGAAAAGGGC‐3′	312–1,128	001002927
PPT	900 bp	Sense: 5′‐TCGAACATGAAAATCCTCGTGGCC‐3′ Antisense: 5′‐CACATCATACAATGACTGAAGACC‐3′	95–994	D17584
PARV	823 bp	Sense: 5′‐TCTGCTCATCCAAGTTGCAG‐3′ Antisense: 5′‐TCCTGAAGGACTCAACCCC‐3′	2–824	NM_013645.2
GABAA‐a1	769 bp	Sense: 5′‐GCCTAATAAGCTCCTGCGTATC‐3′ Antisense: 5′‐CGGTTCTATGGTCGCACTTT‐3′	1,200–1,968	NM_010250.5

### 2.3 Microscopic analysis

#### 2.3.1 Stereological neuron counts

Neuron counts were carried out for striatum and subthalamic nucleus (STN) in Q175 and WT mice. A one‐in‐twelve series of coronal sections immunolabeled for NeuN, DARPP32, PARV, ChAT or nNOS, or stained for cresyl violet was used for type‐specific striatal neuron counts in each mouse, with only one side of the brain analyzed. Typically, this meant that about 10 evenly spaced sections (420 μm apart) were analyzed that spanned the length of the striatum for each striatal marker for each case. In the case of STN, typically six sections were analyzed per animal, from a 1 in 6 series. All counts were performed blinded as to genotype. Neuron counts and volume determinations were obtained using the optical fractionator method with a MicroBrightField Stereo Investigator system (MBF Bioscience, Williston, VT), as in our prior studies (Bu et al., 2016; Guley et al., 2016; Reiner, Lafferty, et al., 2012). In the case of dopaminergic neurons in SNc immunolabeled for TH, the thin shape and variable contours of the SNc from section to section make it problematic to obtain a reliable SNc volume calculation to use for estimating neuron abundance from neuron packing density (Prasad & Richfield, 2008). To avoid these problems, we unilaterally counted every TH+ neuron in SNc in a 1 in 12 series, resulting typically in counts for 4 SNc‐containing sections. We then multiplied by 12 to estimate the total unilateral number of SNc neurons.

#### 2.3.2 Quantification of immunolabeled pallidal neuron types in striatal target areas

We evaluated possible loss of prototypical and arkypallidal neurons of GPe, using immunolabeling for PARV and immunolabeling for FoxP2 to detect these two neuron types, respectively. Additionally, we evaluated loss of PARV+ immunolabeled neurons from GPi and SNr. Finally, we also determined if there was any change in the abundance of GABAA receptors (as reflected in immunolabeling intensity or neuron abundance) on the projection neurons of GPe, GPi, and/or SNr, which use GABA as a neurotransmitter, and all of which (GPi and SNr) or the majority of which (GPe) contain PARV (Glajch et al., 2016; Hegeman et al., 2016; Lee & Tepper, 2007). Note that as part of our evaluation of FoxP2 neurons in GPe, we used immunofluorescence double labeling on fixed WT tissue through GPe to confirm that FoxP2 was restricted to neurons in GPe using double labeling for NeuN and FoxP2, and to confirm that FoxP2 did not occur in PARV+ prototypical GPe neurons using double labeling for PARV and FoxP2, as noted above. For neuron counts in DAB‐immunolabeled tissue, because GPe, GPi, and SNr are all relatively small structures, and because the neuron types of interest are relatively sparse in these structures, it was practical to manually count immunolabeled neurons using a microscope equipped with an eyepiece with a 10 × 10 grid. For GPe and SNr, we estimated neuron abundance by the total unilateral count across all sections in a 1 in 12 series and multiplied by 12 for the total count per structure. In the case of GPi, because of its small size, we bilaterally counted every neuron in it immunolabeled for PARV in a 1 in 6 series of sections, divided the total bilateral count by 2 to determine the mean count per GPi, and then multiplied by 6 to estimate the total number of neurons in a single GPi. For GPe, GPi, and SNr, we additionally determined PARV neuron packing density from a field through the middle of the nucleus for each animal. In the case of GABAA‐α1 immunolabeling in GPe, GPi, and SN, blinded manual counts as described below for ISHH on striatal ENK+ and SP+ neurons were used to determine GABAA‐α1 neuron abundance per unit area of GPe, GPi, and SN. Densitometry was additionally used on the immunolabeled neurons to separately determine their labeling intensity for GABAA‐α1, as described below for densitometry on ISHH‐labeled striatal ENK+ and SP+ neurons. All analysis was conducted blinded to genotype.

#### 2.3.3 Image analysis of immunolabeling in striatal target areas

The abundance of ENK+ fibers and terminals in GPe, of SP+ fibers and terminals in GPi, of SP+ fibers and terminals in SN, and of DARP32+ fibers and terminals in GPe, GPi, and SN was determined using computer‐assisted image analysis, as described previously (Deng et al., 2004; Figueredo‐Cardenas et al., 1994; Meade et al., 2000; Reiner et al., 2007; Sun et al., 2002). For ENK and SP immunolabeling in these striatal target areas, we analyzed a one in six series of sections bilaterally. This meant that we analyzed GPe bilaterally at about 10 closely spaced levels per case, resulting in 20 measurements of GPe. As the GPi is much smaller than GPe, we typically were only able to analyze SP‐immunolabeled fibers in about three closely spaced levels through GPi, resulting in six measurements for GPi. In the case of SP‐immunolabeled fibers in SN, we were again typically able to analyze about 10 closely spaced sections bilaterally, resulting in 20 measurements for SN. For DARPP32 immunolabeling, a one‐in‐twelve series was analyzed, because of limitations on the available tissue due to all of the other markers analyzed. High‐resolution, uniformly illuminated digital images of the immunolabeled sections were acquired using a digital scanner. For each image of each striatal target area used for analysis, the background optical density of the image was adjusted to 50 (on a 0 white–255 black scale) in ImageJ, to standardize the images. For GPe, the nearby internal capsule was used to standardize tissue background, while for GPi and SN, the surrounding cerebral peduncle was used. The structure of interest was then outlined using the polygon tool in ImageJ and the area of the structure and its overall labeling intensity determined. The boundaries of the structures were very clear due to the labeling of the striatal terminals, which are confined to the given striatal target structure. Note that in our SN measurements, however, we analyzed the pars compacta and pars reticulata together, since there are no definitive landmarks in the SP‐immunolabeled tissue to distinguish them. We thus refer to this region as substantia nigra (SN), but it should be noted that given the much greater size of SNr than SNc, the vast majority of the measurement reflects immunolabeled terminals in SNr. Thresholding was next used (as in Deng et al., 2004) to select the immunohistochemically labeled fibers from background, and the percent of the image occupied by the thresholded, labeled fibers was determined, and used as the measure of fiber abundance per target structure. For thresholding, the automatic default threshold function of ImageJ was used once background had been adjusted to 50 grayscale (with 0 = white, and 255 = black). All measurements were carried out blinded with respect to genotype. The striatal fiber abundance in GPe, GPi, and SN was calculated by determining the mean percent of the given structure occupied by labeled fibers for that case, averaged across all sections and sides for that case. This methodology also provided volume data on GPe, GPi, and SN.

#### 2.3.4 Sholl analysis

We previously used Sholl analysis to show that the dendrites of striatal cholinergic interneurons were significantly fewer and shorter in heterozygous Q140 males at 1 and 4 months of age, although there was no reduction in striatal cholinergic interneuron abundance itself (Deng & Reiner, 2016). We further noted that the reduced dendritic branching led to significantly reduced thalamic input to the cholinergic interneurons. Because cholinergic interneurons differentially affect iSPNs versus dSPNs, such reduced thalamic excitatory drive may contribute to early abnormalities in movement in HD, assuming the reduced thalamic excitatory drive occurs in humans as well. Because such an abnormality could also occur for cholinergic interneurons in Q175 mice, irrespective of any cholinergic interneuron loss, we conducted Sholl analysis of camera lucida drawings of randomly selected cholinergic interneurons (at least 20 per case) from the immunolabeled 2‐, 6‐, and 12‐month‐old WT and Q175 cases. The drawing of each neuron was digitized and used for Sholl analysis, which assesses the number of dendritic branch intersections with a series of concentric circles of increasing radii (10 μm steps) from the soma. The Sholl analysis was performed using the Sholl analysis plugin from Fiji (http://fiji.sc/Sholl_Analysis).

#### 2.3.5 NII size and abundance

Sections from mutant mice that had been immunolabeled for ubiquitin were analyzed for NII size and frequency. As WT mice do not form mutant protein aggregates, they were not processed or analyzed for either ubiquitin or EM48 immunolabeling. For each mutant mouse, images were captured using a ×40 objective and analyzed using ImageJ. For cortex, NII size and spatial abundance were determined from images through primary motor cortex (M1), with measurements conducted separately on layers II/III and V‐VI. For NII spatial abundance and size in striatum, measurements were made for central striatum. To determine NII diameter from the images of anti‐ubiquitin immunolabeled sections, NIH ImageJ was used to threshold NIIs. Artifacts in the images were removed prior to thresholding, and the size of the thresholded NIIs determined using the Particle Measurement function. The same thresholding method was also used to determine the spatial abundance of NIIs (i.e., percent of field occupied by NIIs). The anti‐ubiquitin was used in the analysis, since it yielded more prominent immunolabeling than EM48, but colocalizes with EM48 and thus provides suitable information on mutant huntingtin aggregates (Meade et al., 2002).

#### 2.3.6 ISHH—Striatal neuron counts

We counted labeled neurons in fresh‐frozen cryostat‐sectioned tissue that had been labeled by ISHH for SPN types whose perikarya do not label well by immunolabeling, notably ENK+ iSPNs and SP+ dSPNs. Because of the labor‐intensive nature of ISHH labeling, it proved impractical to produce the multiple closely spaced series of labeled sections needed for stereology. We thus used an alternative approach involving stereological principles—we determined the abundance of ENK+ iSPNs and SP+ dSPNs in striatum by separately determining for each case the volume of striatum and the number of ENK+ perikarya per unit volume of striatum or the number of SP+ perikarya per unit volume of striatum. Striatal volume was determined by measuring striatal area unilaterally for each ISHH case in an evenly spaced series of cresyl violet‐stained cryostat sections (typically every tenth, resulting in 15–20 sections measured), and determining volume from these areal measurements, section thickness (20 μm), section abundance, and section spacing. We counted neurons in a single standard field in central striatum at a level just anterior to the anterior commissure. We also used ImageJ to measure the optical density of the labeling at this level. For these intensity measurements, striatal background was set to zero by subtracting background intensity, the striatum was circumscribed to select it, and the optical density of the entire striatum measured. Additionally, labeled neurons in the entire striatum were selected by thresholding using the default automatic threshold function, and the specific labeling intensity determined. These assessments were performed on digital images captured using a high‐resolution Aperio Scanscope XT Scanner. All assessments were done in a blinded fashion as to genotype, although using autothreshold controlled for bias as well.

#### 2.3.7 Quantification of ISHH labeling of pallidal neuron types

We also evaluated the spatial abundance and labeling intensity of neurons in GPe, GPi, and SNr containing PARV and GABAA‐α1 as detected by ISHH. This provided an opportunity to evaluate changes at the transcript level that might not be reflected at the level of protein as detected by IHC. For these studies, cryostat sections were used as in the case of ENK‐ISHH and SP‐ISHH, and sections through the striatal target areas from the same cases were used. For intensity measurements, background was set to zero, the region of interest (GPe, GPi, or SN) was circumscribed to select it, and the optical density of the entire region measured. Then, labeled neurons were selected by thresholding using the default automatic threshold function, and the specific labeling intensity of the neurons determined. A count of labeled neurons per unit area was also obtained. Matched levels through the midpoint of GPe, GPi, or SN were used for analysis across cases. The analysis was conducted blinded as to genotype.

#### 2.3.8 Statistics

In general, and unless stated otherwise, comparisons were made between WT and mutant at each age by unpaired, two‐tailed *t*‐tests. For some endpoints, as noted on a case by case basis in the Results section, ANOVA was used to compare WT and mutant across age. The relationship of various neurochemical parameters to behavioral outcomes, and of mutant protein aggregate abundance to behavioral and neurochemical outcomes was assessed using regression analysis.

## 3 RESULTS

### 3.1 Behavioral studies

#### 3.1.1 Body weight

**FIGURE 1:**
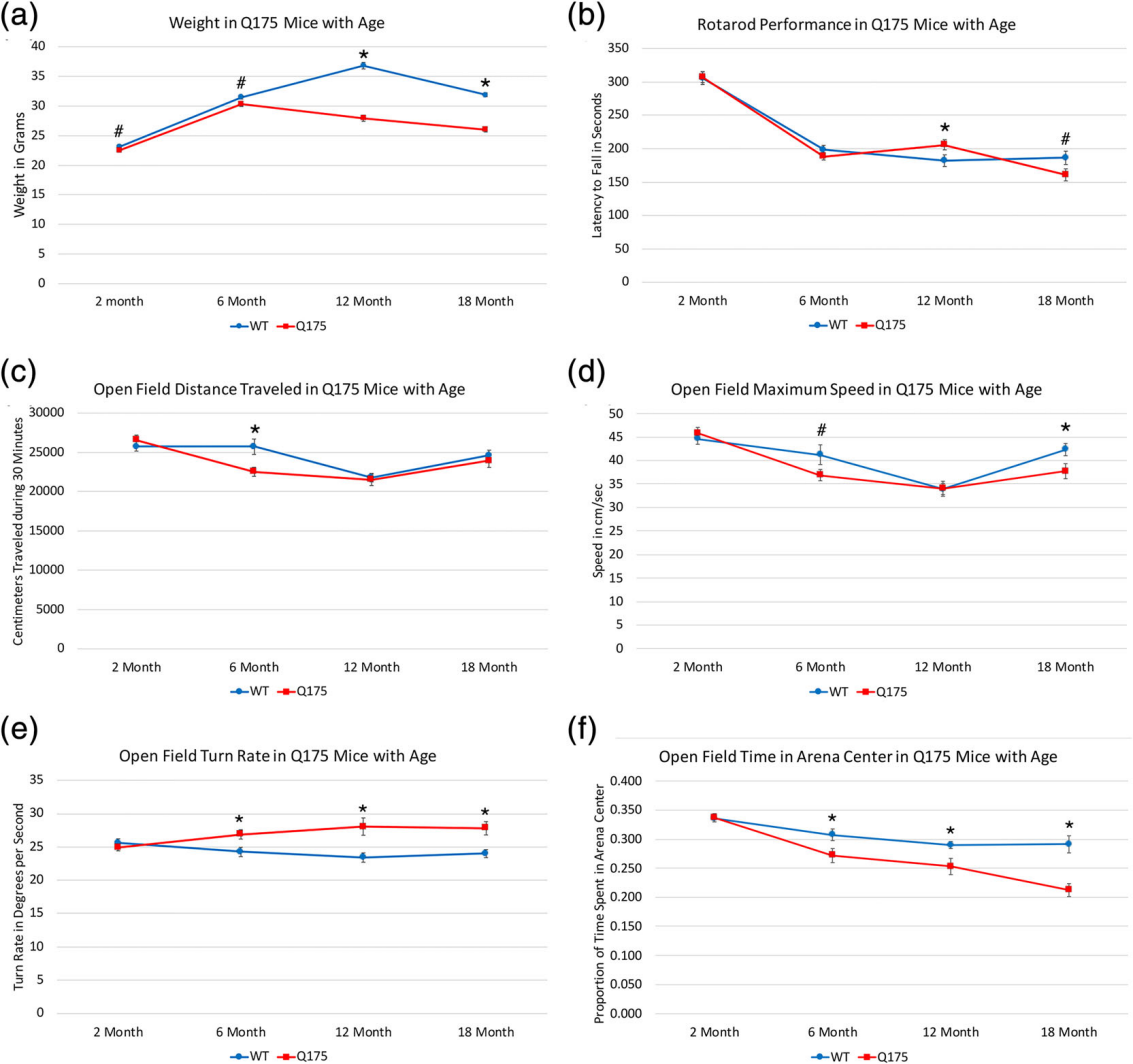
Graphs showing the progression in weight and behavioral endpoints with age in heterozygous male Q175 mice compared to age‐matched male WT mice. (a) Q175 weight was slightly but not significantly less already at 6 months and then diverged further from WT weight with age to 12 and 18 months. (b) Rotarod performance (latency to fall) did not differ between mutant and WT at 2 or 6 months of age, but was significantly better in Q175 at 12 months, but trended toward significantly worse at 18 months by *t*‐test. (c) Open field distance traveled did not differ between mutant and WT, except at 6 months, when Q175 performed significantly more poorly. (d) Like for distance, open field maximum speed did not differ between mutant and WT at 2 and 12 months, but Q175 trended toward poorer performance at 6 months and were significantly worse at 18 months. (e) Open field turn rate was significantly increased in Q175 mice at 6, 12, and 18 months. An increase in turn rate appears to reflect hyperkinesia. (f) Open field time spent moving in the arena center was significantly reduced in Q175 mice at 6, 12, and 18 months, reflecting heightened anxiety at these ages. Asterisks indicate significant differences by *t*‐test between Q175 and age‐matched WT, and the pound symbols indicate trends toward significance. Animal numbers: 2m WT *n* = 30, 2m Q175 *n* = 30; 6m WT *n* = 30, 6m Q175 *n* = 30; 12m WT *n* = 30, 12m Q175 *n* = 29; 18m WT *n* = 27, 18mQ175 *n* = 25

The WT versus Q175 body weight difference was significant at 12 and 18 months (Figure 1a), but not at 2 or 6 months, although Q175 weight trended toward less than WT in 2‐ and 6‐month‐old Q175 mice (Table 1). The difference was small at 2 months (−2.6%) and 6 months of age (−3.6%), but much larger at 12 months of age (−24.1%) and at 18 months of age (−18.5%). Q175 weight increased from 2 to 6 months, but declined progressively from 6 to 18 months of age for Q175 mice. WT mice, in contrast, showed progressive increase from 2 to 12 months, and a slight decline thereafter.

#### 3.1.2 Rotarod performance

Rotarod performance was not significantly different between the WT and Q175 mice at 2 or 6 months of age (Figure 1b). Somewhat surprisingly, the Q175 mice at 12 months remained on rotarod 13.1% longer than WT mice and this difference was significant (*p* = .0447). As we have observed that rotarod performance in normal mice is inversely linked to body weight, we believe it likely that the better performance by mutant mice at 12 months of age is related to their considerably lesser body weight than shams. In contrast, in 18‐month‐old Q175 mice, we observed poorer rotarod performance (13.7% less) than in WT mice. For the average of the three test session trials, this difference trended toward significance (*p* = .062), and a consistent reduction in the mutants across all three test trials was significant by one‐way ANOVA (*p* = .0059). The emergence of a rotarod deficit by 18 months of age despite the yet lesser weight of the mutant mice than at 12 months is likely to be a consequence of the greater neural decline, as detailed below, evident by 18 months of age.

#### 3.1.3 Open field performance

The Q175 mice at 6 months showed a significant 12.4% reduction in distance traveled in open field (*p* = .00681), but a reduction in distance traveled was not seen at either 2 months or, somewhat surprisingly, the later ages (Figure 1c). The Q175 mice at 6 months also showed a trend toward reduction in maximum speed (10.6% decrease, *p* = .07150) that was not evident at 2 months (*p* = .42666) or 12 months (*p* = .97959) in Q175 mice, but was significant in 18‐month‐old Q175 mice (10.9% decrease, *p* = .02611; Figure 1d). Notably, we also saw a significant 10.9% increase in turn rate in the Q175 mice at 6 months (*p* = .01115), but none at 2 months (*p* = .54671), and the increase in turn rate remained significantly greater in the mutant mice than in WT at 12 months (19.9% increase, *p* = .00190) and 18 months (15.7% increase, *p* = .00133). We interpret the increased turn rate as a hyperkinetic behavior, suggesting Q175 mice show early onset and persistence to at least 18 months of hyperkinesia (Figure 1e). The Q175 mice at 6 months also showed a significant reduction in open field arena center occupancy during movement (11.6% decrease, *p* = .02902) that was also seen at 12 months (12.7% decrease, *p* = .01917), and 18 months (27.0% decrease, *p* = .00010), but not at 2 months (*p* = .86461; Figure 1f). Reduced center occupancy in open field is generally considered indicative of increased anxiety. Thus, anxiety and hyperkinesia seem to be persistent and early appearing behavioral abnormalities in male heterozygous Q175 mice. Note that numerous other endpoints assessed by our open field system did not show statistically significant effects of the Q175 genotype that were consistent across age. These included various measures related to the length, duration, and spatial distribution of progression segments, to the speed, duration, and spatial distribution of lingering episodes, acceleration, to stops, to stamina, and to progression segment path curvature. Progression segment length did, however, show trends toward reduction in Q175 mice at 6 and 18 months.

### 3.2 Striatal neurons

#### 3.2.1 iSPN perikarya

**FIGURE 2:**
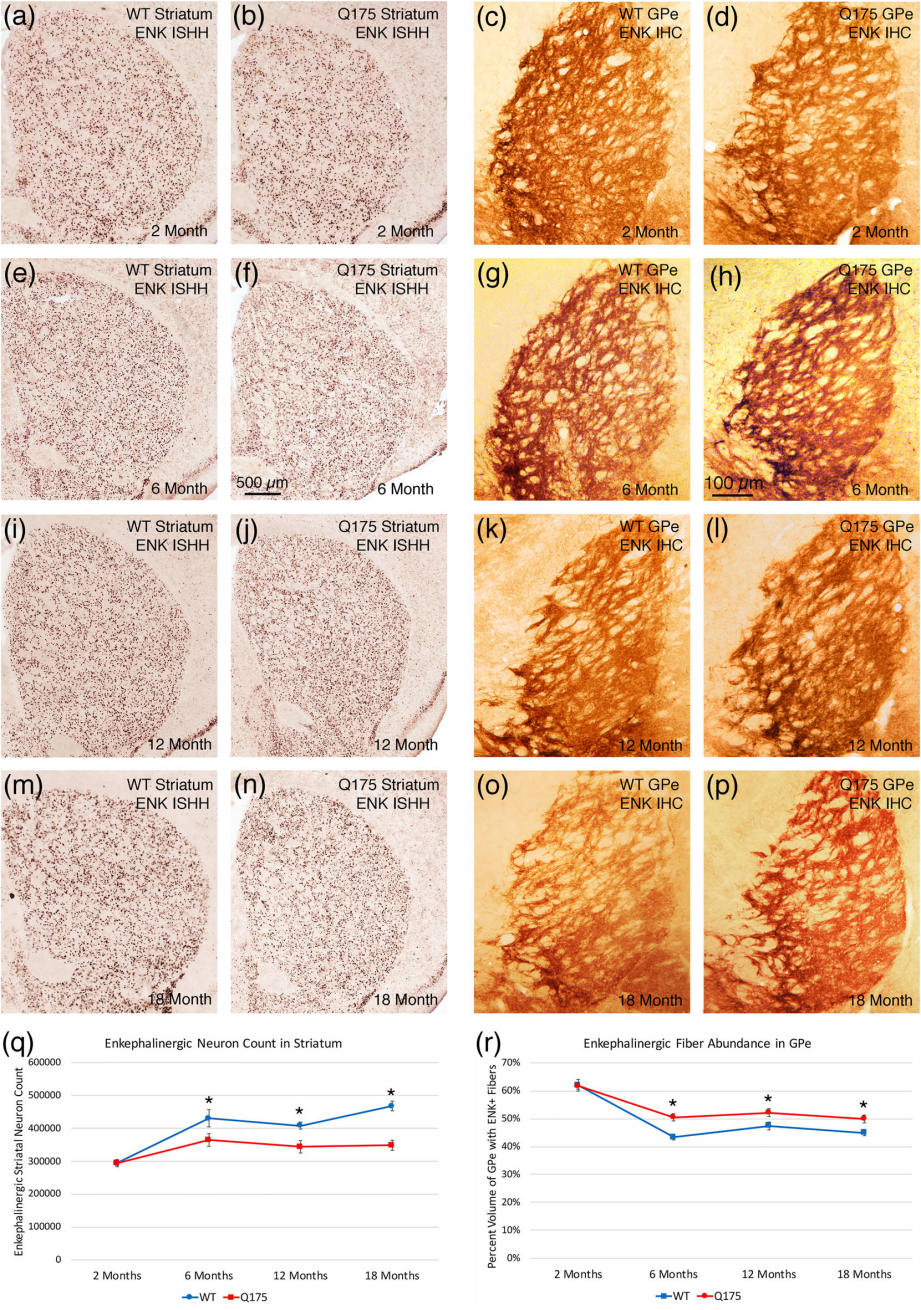
Images showing ENK ISHH in striatum of WT and Q175 mice (a, b, e, f, i, j, m, n), images showing ENK immunolabeling of striatal terminals in GPe of WT and Q175 mice (c, d, g, h, k, l, o, p), graph showing striatal ENK neuron abundance in WT and Q175 mice (q), and graph showing immunolabeled ENK+ striato‐GPe fiber abundance in WT and Q175 mice (r), in each case at 2, 6, 12, and 18 months. Note that ENK+ striatal neuron abundance and labeling intensity is less in Q175 than WT at 6, 12, and 18 months of age, but that ENK+ striato‐GPe fiber abundance is increased at these same ages. The graph of ENK+ striatal neuron abundance (r) shows that ISHH ENK+ striatal neuron abundance is significantly decreased, somewhat progressively, at 6, 12, and 18 months of age, while the graph of ENK+ striato‐GPe fiber abundance (q) shows that ENK+ striato‐GPe fibers are significantly more abundant at these same ages. The scale bar in (f) pertains to all ISHH images, while that in image (h) pertains to all immunolabeling images. All images are from sections in the coronal plane, with dorsal to the top and medial to the left. Asterisks indicate significant difference by *t*‐test between Q175 and age‐matched WT. Animal numbers for ISHH: 2m WT *n* = 9, 2m Q175 *n* = 9; 6m WT *n* = 10, 6m Q175 *n* = 9; 12m WT *n* = 10, 12m Q175 *n* = 9; 18m WT *n* = 10, 18m Q175 *n* = 10. Animal numbers for immunohistochemical labeling (IHC): 2m WT *n* = 10, 2m Q175 *n* = 10; 6m WT *n* = 10, 6m Q175 *n* = 10; 12m WT *n* = 10; 12m Q175 *n* = 9; 18m WT *n* = 11, 18m Q175 *n* = 9

The ISHH revealed a significant, substantial, and early‐onset reduction in message for individual ENK neurons in Q175 mice—30.3% less than WT at 6 months (*p* = .00667), 9.6% less at 12 months (*p* = .00489), and 8.7% less at 18 months (*p* = .03692; Figure 2). Moreover, a small but significant 4.6% reduction in signal per neuron was already evident at 2 months (*p* = .01452). The reduction in signal intensity for ENK neurons tended to be greater in medial than lateral striatum. Additionally, neuron counts in the ISHH material revealed significantly fewer ENK neurons in Q175 mice than WT mice at all three older ages, 15.3% fewer at 6 months (*p* = .03584), 15.8% fewer at 12 months (*p* = .00701), and 25.5% fewer at 18 months (*p* = .000017), but not at 2 months (*p* = .94546). As discussed in more detail below in the section on overall striatal neuron counts, despite the reduction in ENK+ iSPNs, stereological pan‐striatal neuron counts showed no overall striatal neuron loss per se in Q175 mice. Thus, the reduction in ENK+ iSPNs appears to reflect ENK message loss, rendering some ENK neurons undetectable, rather than ENK neuron loss.

#### 3.2.2 iSPN fibers in GPe

We performed blinded densitometric analysis of ENK immunolabeling in GPe. Given the reduced ENK expression in iSPNs, it was a surprise to see a significant increase in ENK striato‐GPe terminals in Q175 mice in our immunohistochemical analyses at 6 months of age (16.4% more than in WT, *p* = .00054), 12 months of age (10.1% more than in WT, *p* = .02771), and 18 months of age (10.9% more than in WT, *p* = .01759; Figure 2). ENK striato‐GPe terminals in Q175 mice were, however, no different than in WT at 2 months (*p* = .71062).

#### 3.2.3 Correlation of iSPN traits with behavior

Regression analysis showed that striatal and/or striato‐GPe ENK levels were significantly correlated with the abnormalities in multiple behavioral parameters. For example, across the WT and Q175 cases at 6, 12, and 18 months of age, the abundance of ENK‐ISHH perikarya counted in striatum was significantly correlated with distance traveled in open field (*r* = .459) and the length of progression segments in open field (*r* = .356), meaning fewer ENK perikarya led to less distance traveled in open field and shorter units of progression. Moreover, the abundance of ENK‐ISHH perikarya counted in striatum across the WT and Q175 cases at 6, 12, and 18 months of age was significantly inversely correlated with turn rate (*r* = −.392), meaning the reduction in ENK neurons in Q175 mice was associated with increased turning. At 6 months of age, when speed was seen to be reduced in the mutant mice, the abundance of ENK‐ISHH perikarya was highly and significantly correlated with maximum speed (*r* = .796) in open field, suggesting the lessening of ENK‐labeled neurons in Q175 mice also led to slowing. The linking of ENK neuron reduction to increased turning, a hyperkinetic behavior, is of interest, as hyperkinesia is the outcome predicted for striatal ENK neuron loss/hypofunction in the indirect–direct pathway model of basal ganglia function (Albin, Young, & Penney, 1989; DeLong, 1990; Reiner et al., 1988). Note, however, that the reduction in ENK‐labeled perikarya was also associated with such hypokinetic signs as lessened distance traveled and slowing. At 18 months, when a rotarod deficit was seen, regression analysis showed that striato‐GPe ENK fiber abundance was significantly inversely correlated (−0.463) with rotarod performance. Thus, the higher level of striato‐GPe ENK as seen in the mutants (reflective of iSPN dysfunction) was significantly associated with poorer rotarod performance.

#### 3.2.4 dSPN Perikarya

**FIGURE 3:**
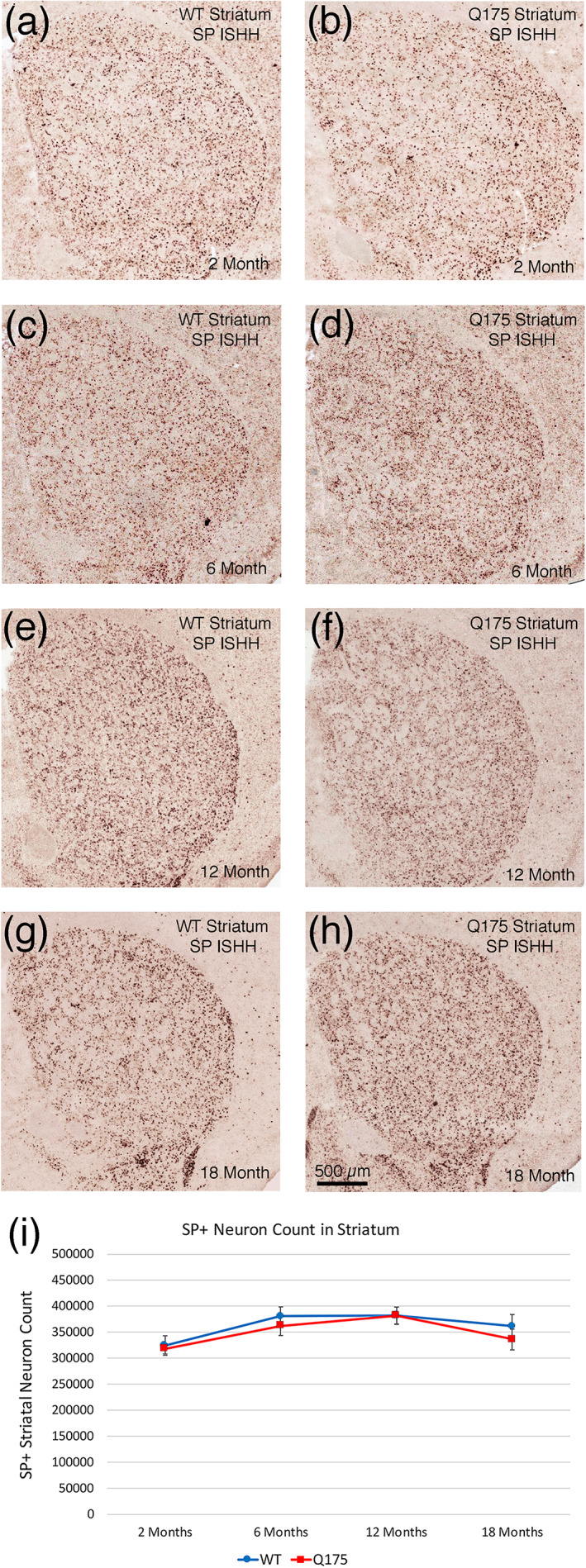
Images showing SP ISHH in striatum of WT and Q175 mice (a–h), and graph showing striatal SP neuron abundance in WT and Q175 mice (i), in each case at 2, 6, 12, and 18 months. Note that SP+ striatal neuron abundance and labeling intensity is similar in Q175 and WT at all ages. The graph of SP+ striatal neuron counts confirms that ISHH SP+ striatal neuron abundance is similar in Q175 and WT at all ages. The scale bar in (h) pertains to all ISHH images. All images are from sections in the coronal plane, with dorsal to the top and medial to the left. Animal numbers for ISHH: 2m WT *n* = 10, 2m Q175 *n* = 10; 6m WT *n* = 7, 6m Q175 *n* = 8; 12m WT *n* = 10, 12m Q175 *n* = 10; 18m WT *n* = 10, 18m Q175 *n* = 10

There was no change in the signal intensity or abundance of detectible SP+ striatal perikarya in Q175 mice over the 2‐ to 18‐month period. The SP signal intensity of individual perikarya showed no significant difference between mutant and WT mice at any age—mutant mice were 98.5% of WT at 2 months (*p* = .63957), 98.2% at 6 months (*p* = .30828), 92.4% at 12 months (*p* = .20121), and 114.2% at 18 months (*p* = .21573; Figure 3). Neurons counts also showed no significant difference between mutant and WT mice in the abundance of SP+ dSPNs at 2 months (98.0% of WT, *p* = .76389), 6 months (95.0% of WT, *p* = .23511), 12 months (99.9% of WT, *p* = .98738), or 18 months (93.0% of WT, *p* = .20676; Figure 3).

#### 3.2.5 dSPN fibers in GPi and SN

**FIGURE 4:**
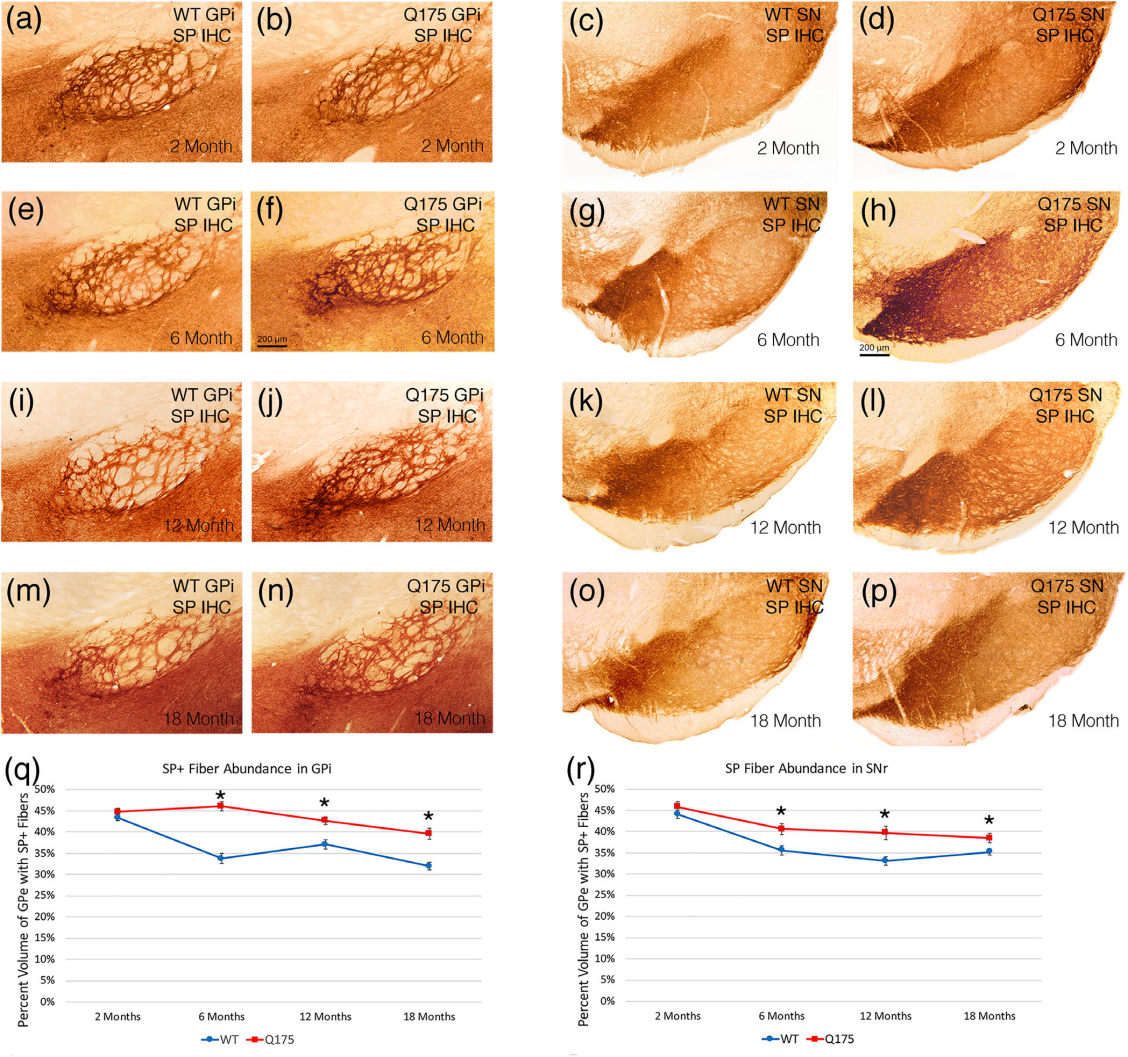
Images showing SP immunolabeling of striatal terminals in GPi of WT and Q175 mice (a, b, e, f, i, j, m, n), images showing SP immunolabeling of striatal terminals in SN in WT and Q175 mice (c, d, g, h, k, l, o, p), graph showing abundance of SP immunolabeled striato‐GPi terminals in WT and Q175 mice (q), and graph showing SP+ striato‐SN terminal abundance in WT and Q175 mice (r), in each case at 2, 6, 12, and 18 months. Note that SP+ striatal fiber abundance in GPi and SN is increased in Q175 mice at 6, 12, and 18 months. The graphs show quantification of these results. The scale bar in (f) pertains to all striato‐GPi images, while that in (h) pertains to all striato‐SN images. All images are from sections in the coronal plane, with dorsal to the top and medial to the left. Asterisks indicate significant difference by *t*‐test between Q175 and age‐matched WT mice. Animal numbers for GPi and SN IHC: 2m WT *n* = 10, 2m Q175 *n* = 10; 6m WT *n* = 10, 6m Q175 *n* = 10; 12m WT *n* = 10, 12m Q175 *n* = 9; 18m WT *n* = 11, 18m Q175 *n* = 9

Although the ISHH did not detect any significant abnormalities in SP expression by dSPNs in the mutant mice, the immunolabeling analysis of the abundance of SP+ fibers in GPi and SN revealed that dSPNs were abnormal in that they showed a statistically significant buildup of SP+ terminals in GPi and in SN at the three older ages—136.4% of WT for striato‐GPi (*p* = .0000006) and 114.0% of WT for striato‐SN (*p* = .00141) at 6 months, 115.1% of WT for striato‐GPi (*p* = .00106) and 119.9% of WT for striato‐SN (*p* = .00127) at 12 months, and 123.8% of WT for striato‐GPi (*p* = .00023) and 109.4% of WT for striato‐SN (*p* = .02517) at 18 months (Figure 4). No such differences were seen at 2 months (GPi, *p* = .23149; SN, *p* = .28784; Figure 4).

#### 3.2.6 Correlation of dSPN traits with behavior

Despite the absence of SP+ neuron reduction in the Q175 mice, regression analysis showed that the abundance of SP+ striatal neurons across WT and mutant mice at 6 months was significantly inversely correlated with stops per unit distance (*r* = −.621). Thus, the overall results show the importance of SP+ dSPNs for maintaining progression in open field, as fewer SP+ dSPNs led to a diminished ability to progress (i.e., more stops). Regression analysis at 18 months, showed that striato‐GPi SP levels were inversely correlated with rotarod performance (−0.455). Thus, the higher striato‐GPi SP fiber abundance in the mutants was significantly associated with poorer rotarod performance, suggesting that a dysfunctional striato‐GPi dSPNs, as reflected in abnormally elevated SP+ fiber abundance in GPi, may have contributed to impaired motor performance.

#### 3.2.7 DARPP32 perikarya

**FIGURE 5:**
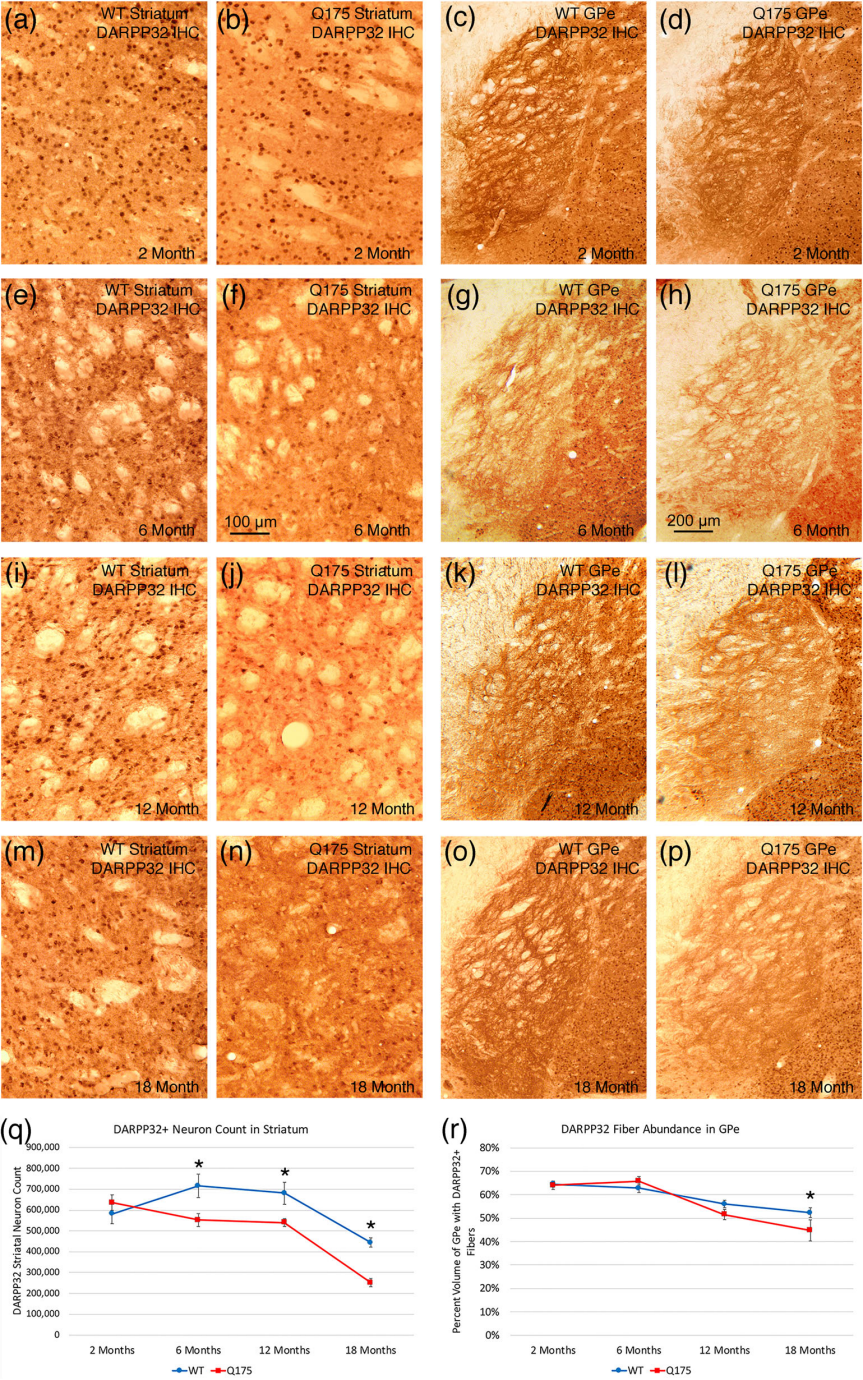
Images showing DARPP32 immunolabeling of striatal perikarya in WT and Q175 mice (a, b, e, f, i, j, m, n), images showing DARPP32 immunolabeling of striatal terminals in GPe in WT and Q175 mice (c, d, g, h, k, l, o, p), graph showing abundance of DARPP32 immunolabeled striatal perikarya in WT and Q175 mice (q), and graph showing DARPP32+ striato‐GPe terminal abundance in WT and Q175 mice (r), in each case at 2, 6, 12, and 18 months. Note that DARPP32+ striatal neuron abundance is reduced in Q175 at 6, 12, and 18 months, and DARPP32+ striato‐GPe fibers are reduced at 18 months. The graphs show quantification of these results. The scale bar in (f) pertains to all striatal images, while that in (h) pertains to all striato‐GPe images. Asterisks indicate significant difference by *t*‐test between Q175 and age‐matched WT. All images are from sections in the coronal plane, with dorsal to the top and medial to the left. Animal numbers for striatum: 2m WT *n* = 10, 2m Q175 *n* = 10; 6m WT *n* = 10, 6m Q175 *n* = 9; 12m WT *n* = 10, 12m Q175 *n* = 9; 18m WT *n* = 10, 18m Q175 *n* = 7. Animal numbers for GPe: 2m WT *n* = 10; 2m Q175 *n* = 10; 6m WT *n* = 10, 6m Q175 *n* = 9; 12m WT *n* = 10, 12m Q175 *n* = 9; 18m WT *n* = 10, 18m Q175 *n* = 6

DARPP32 is found in both iSPNs and dSPNs (and thus should be present in about 95% of striatal neurons), but in mice, we have found it is only detectible by immunolabeling in about 35% of SPNs, apparently due to a limitation in primary antibody penetration (Figure 5). In any case, counts of DARPP32‐immunolabeled SPNs showed a progressive reduction in Q175 mice compared to age‐matched WT after 2 months of age—a significant 22.9% reduction (*p* = .01950) at 6 months, a significant 20.6% reduction (*p* = .02939) at 12 months, and a significant 43.3% reduction (*p* = .00002) at 18 months. No significant reduction was seen at 2 months of age (*p* = .37718). The reductions at 6, 12, and 18 months appears to reflect loss of DARPP32 expression rather than SPN loss, since (as discussed in more detail below) stereological counts of cresyl violet‐stained striatal neurons and NeuN‐immunolabeled neurons showed there was no significant overall neuron loss in striatum per se.

#### 3.2.8 DARPP32 fibers in striatal target areas

**FIGURE 6:**
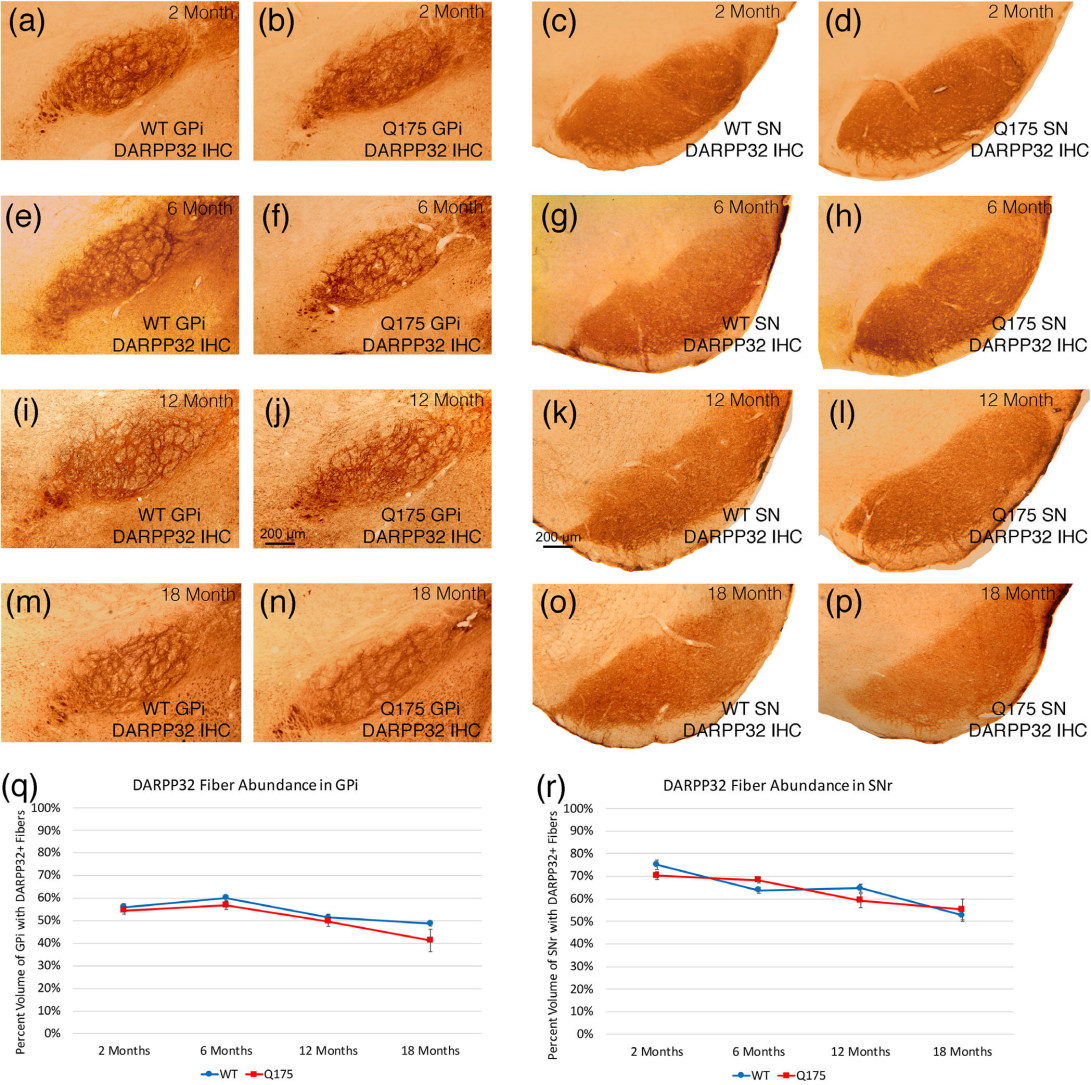
Images showing DARPP32 immunolabeling of striatal terminals in GPi of WT and Q175 mice (a–d, i, j, m, n), images showing DARPP32 immunolabeling of striatal terminals in SN of WT and Q175 mice (c, d, g, h, k, l, o, p), graph showing abundance of DARPP32 immunolabeled striato‐GPi terminals in WT and Q175 mice (q), graph showing DARPP32+ striato‐SN terminal abundance in WT and Q175 mice (r), in each case at 2, 6, 12, and 18 months. Note that DARPP32+ striatal fiber abundance in GPi and SN typically does not differ significantly between WT and Q175 mice at any age. The graphs show quantification of these results. The scale bar in (j) pertains to all striato‐GPi images, while that in (k) pertains to all striato‐SN images. All images are from sections in the coronal plane, with dorsal to the top and medial to the left. Animal numbers for GPi: 2m WT *n* = 10, 2m Q175 *n* = 10; 6m WT *n* = 10, 6m Q175 *n* = 6; 12m WT *n* = 10, 12m Q175 *n* = 9; 18m WT *n* = 10, 18m Q175 *n* = 6. Animal numbers for SN: 2m WT *n* = 10, 2m Q175 *n* = 10; 6m WT *n* = 5, 6m Q175 *n* = 6; 12m WT *n* = 10, 12m Q175 *n* = 9; 18m WT *n* = 10, 18m Q175 *n* = 6

The DARPP32 immunolabeling of terminals in striatal target areas also provided evidence for a differential effect on the SPN subtypes of the mutation as Q175 mice aged (Figures 5 and 6). At 2 and 6 months of age, no significant difference between WT and Q175 mice was seen for the abundance of DARPP32‐immunolabeled fibers in GPe (Figure 5). At 12 months of age, however, we saw a trend toward a reduction in DARPP32+ fiber abundance in GPe (8.1%), and at 18 months of age, we observed a significant 14.6% reduction in DARPP32+ fibers in GPe (*p* = .03354; Figure 5). For the GPi, the DARPP32‐immunolabeled fibers were not significantly different from WT at any of the four ages examined (Figure 6). In the case of nigra as well, the DARPP32‐immunlabeled fibers were not significantly different from WT at 2, 6, 12, or 18 months of age (Figure 6). Thus, our DARPP32 data appears to show a preferential and progressive reduction in expression in striatal DARPP32+ iSPNs projecting to GPe, but not for dSPNs projecting to GPi or nigra.

#### 3.2.9 Correlation of DARPP32 traits with behavior

Striatal DARPP32 neuron abundance across the WT and Q175 cases at 6, 12, and 18 months of age was significantly correlated with distance traveled in open field (*r* = .292) and maximum speed in open field (*r* = .266), meaning fewer DARPP32+ perikarya led to less distance traveled in open field and slower progression. Moreover, the abundance of DARPP32+ perikarya in striatum across the WT and Q175 cases at 6, 12, and 18 months of age was significantly inversely correlated with turn rate (*r* = −.354), meaning the reduction in DARPP32+ neurons in Q175 mice was associated with increased turning, the hyperkinetic behavior also associated with reduced ENK striatal neurons. At 18 months, both the loss of striatal DARPP32+ neurons and the loss of DARPP32+ fibers in GPe were significantly inversely correlated with the large increase in turn rate (DARRP32+ neuron abundance with turn rate, *r* = −.504; and DARPP32+ fiber abundance in GPe with turn rate, *r* = −.586). This increase in turning with fewer DARPP32 neurons and fewer DARPP32 striato‐GPe fibers reflects a hyperkinesia that would be expected from iSPN dysfunction or loss, based on the standard direct–indirect pathway model of basal ganglia function (Albin et al., 1989; DeLong, 1990; Reiner et al., 1988).

#### 3.2.10 Overall striatal neuron abundance

**FIGURE 7:**
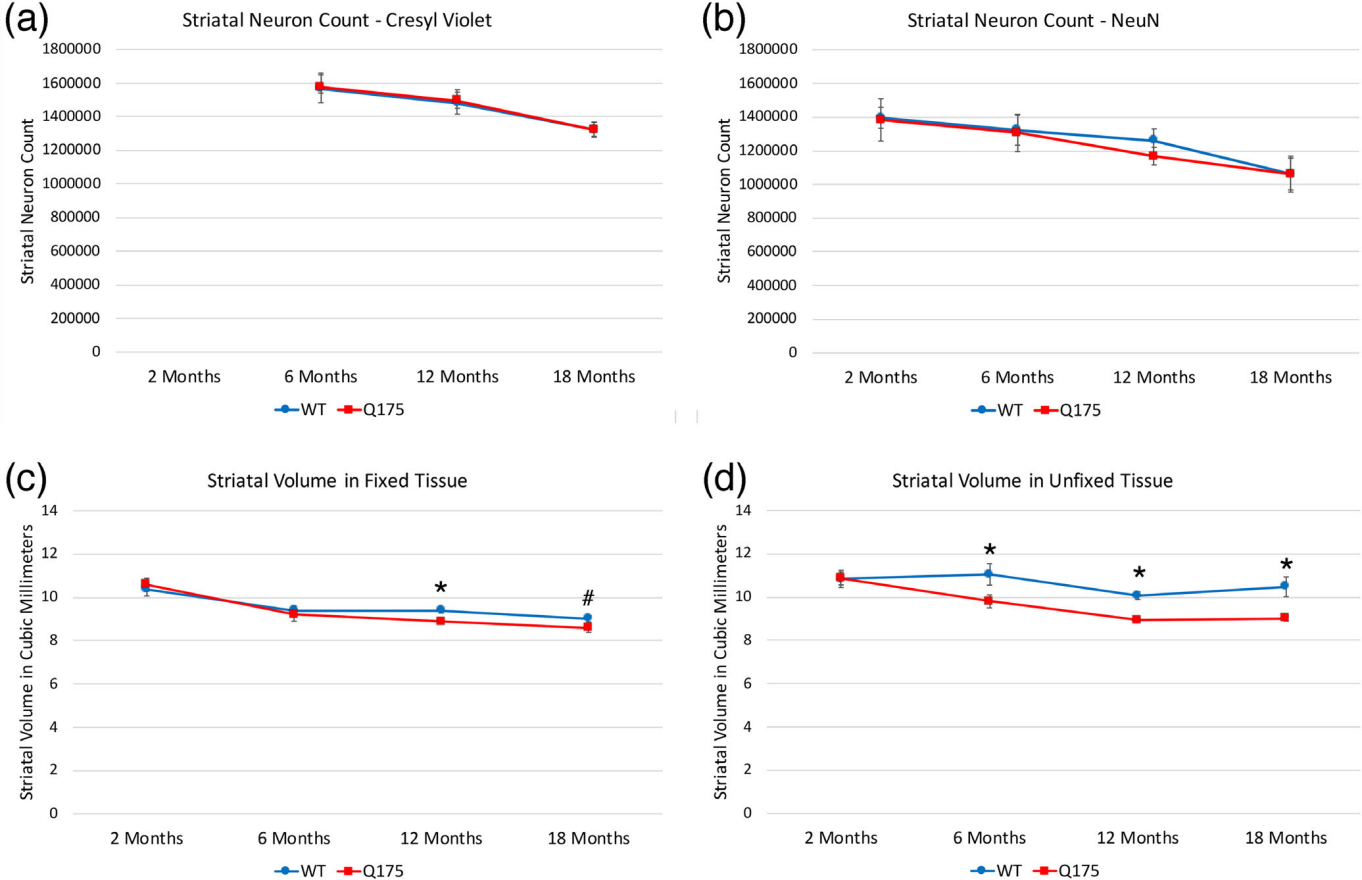
Stereological counts of striatal neurons stained for cresyl violet in WT and Q175 mice at 6, 12, and 18 months of age (a), stereological counts of striatal neurons immunostained for NeuN in WT and Q175 mice at 2, 6, 12, and 18 months of age (b), mean striatal volume in fixed tissue across all stained tissue in WT and Q175 mice at 2, 6, 12, and 18 months of age (c), and mean striatal volume in unfixed tissue used for ISHH in WT and Q175 mice at 2, 6, 12, and 18 months of age (d). WT and Q175 mice do not differ in striatal neuron abundance, as determined for both cresyl violet‐stained tissue and NeuN‐immunostained tissue. Striatal volume in fixed tissue did not differ between mutant and WT at 2 or 6 months, but the mean for all markers did differ slightly but significantly at 12 months (asterisk) and the pooled markers also showed a significant difference at 18 months (pound symbol). Thus, fixed striatum was slightly smaller in mutants at 12 and 18 months. In contrast, unfixed striatum was significantly (asterisks) about 10–15% less in volume in mutants at 6, 12, and 18 months of age. Animal numbers for cresyl violet counts: 6m WT *n* = 10, 6m Q175 *n* = 10; 12m WT *n* = 10, 12m Q175 *n* = 9; 18m WT *n* = 11, 18m Q175 *n* = 9. Animal numbers for NeuN counts: 2m WT *n* = 5, 2m Q175 *n* = 5; 6m WT *n* = 10, 6m Q175 *n* = 9; 12m WT *n* = 10, 12m Q175 *n* = 9; 18m WT *n* = 6, 18m Q175 *n* = 6. Animal numbers for fixed tissue volume: 2m WT *n* = 10, 2m Q175 *n* = 10; 6m WT *n* = 10, 6m Q175 *n* = 10; 12m WT *n* = 10, 12m Q175 *n* = 9; 18m WT *n* = 11, 18m Q175 *n* = 9. Animal numbers for unfixed tissue volume: 2m WT *n* = 10, 2m Q175 *n* = 9; 6m WT *n* = 9, 6m Q175 *n* = 10; 12m WT *n* = 10, 12m Q175 *n* = 10; 18m WT *n* = 6, 18m Q175 *n* = 7

Our stereological analysis did not detect a difference in cresyl violet‐stained neuron abundance between WT and mutant at 6, 12, or 18 months of age (Figure 7). Our stereological analysis of NeuN‐immunolabeled striatal neurons confirmed our findings using cresyl violet‐stained material—there was no loss of striatal neurons, although the NeuN counts were slightly less than the cresyl violet counts for any given group. Whether this reflects a greater sensitivity of cresyl violet in neuron detection or an inadvertent inclusion of some labeled glia in the counts (or both) is uncertain. In any case, these findings indicate that the reductions seen for ENK and DARPP32 perikarya in Q175 striatum appear to reflect neuronal dysfunction and diminished expression of these substances, rather than loss of neurons that contain them per se. Although our stereological analysis did not detect a difference in cresyl violet‐stained neuron abundance between WT and Q175 mice at any age examined, the counts in both WT and Q175 mice at 6 months (Figure 7) were slightly more than in the genotyped‐matched mice at 12 and 18 months of age. In general, for both genotypes, cresyl violet‐stained striatal neurons declined about 5% from 6 to 12 months, and another 10% between 12 and 18 months. One‐way ANOVA confirmed a significant effect of age on the counts of cresyl violet‐stained neurons in striatum for both the WT (*p* = .038817) and Q175 mice (*p* = .000419). Post hoc comparisons using the Bonferroni correction for multiple comparisons confirmed that the declines from 6 to 18 months were significant for both WT (*p* = .039026) and Q175 mice (*p* = .000337). Notably, the comparisons further showed that the cresyl violet stained striatal neuron abundance at 18 months was not significantly less than at 12 months for WT mice (*p* = .285896), but was for Q175 mice (*p* = .016428). Thus, there may be an age‐related progressive striatal neuron decline from 6 to 12 to 18 months that is slightly compounded by the mutant genotype from 12 to 18 months. Similar decline from 6 to 18 months was also seen in the counts of NeuN‐immunostained striatal neurons, but in this case, the decline did not differ significantly between WT and Q175 mice.

#### 3.2.11 Striatal volume—Fixed tissue

In our stereological studies of 2‐month‐old mice, no significant differences were seen between WT and Q175 mice in striatal volume for fixed tissue (from NeuN‐immunostained and DARPP32 immunostained tissue; Figure 7). In our stereological studies in 6‐month‐old mice of the fixed immunolabeled (NeuN, DARPP32, PARV, and ChAT) and cresyl violet‐stained tissue (Figure 7), we found that mean striatal volume for the fixed tissue was not significantly less in Q175 than in WT mice for the cresyl violet stained tissue or the ChAT, PARV or DARPP32 immunolabeled tissue, but was for the NeuN immunolabeled tissue (9.2% less than WT; *p* = .03696). Averaging these values together yielded a 2.5% lessening in mutants that was not significant. In the case of cresyl violet‐stained or immunolabeled tissue from 12‐month‐old mice, Q175 striatal volume ranged from about 3–7% less, and was significantly less in the case of the DARPP32 immunolabeled tissue (6.2% less) and ChAT immunolabeled tissue (5.0% less), and for all labeling methods averaged together (4.9% less; *p* = .03408). At 18 months, we also observed a consistently lesser striatal volume in the Q175 mice, which ranged, depending on the stain, from 2.5% less (DARPP32 immunolabeling) to 9.7% less (ChAT immunolabeling). Averaging all estimates of striatal volume obtained from stereological analysis of the fixed tissue (cresyl violet, NeuN, DARPP32, ChAT, PARV, and nNOS) at 18 months, we observed a 4.7% lesser volume in Q175 mice, which was not significantly different between WT and Q175 (*p* = .10937). Pooling rather than averaging all WT volume measures and comparing to all pooled Q175 volume measures, however, yielded a significantly lower striatal volume for Q175 mice (4.9% less, *p* = .04011). Given the consistency across markers and the pooled values, these results suggest that striatal volume appears about 5% less in the fixed Q175 tissue at 12 and 18 months of age, but is not substantially different at the earlier ages.

#### 3.2.12 Striatal volume—Fresh frozen tissue

We found that striatal volume in the fresh frozen tissue used for ISHH was greater than for the age‐matched fixed tissue used for cresyl violet or immunolabel analysis (Figure 7) for both genotypes, apparently reflecting the brain shrinkage caused by dehydration during fixation and tissue processing. Notably, the difference between fixed and unfixed tissue was considerably greater in the case of WT mice than the Q175 mice. As a result, the Q175 striatal volume was typically at least 10% less than in WT in the fresh frozen tissue, at least beyond 2 months of age. For example, striatal volume in the fresh frozen Q175 tissue for 6‐month‐old mice was 11.1% less than in WT, a significant difference between mutant and WT (*p* = .04541). Similar results were observed for the fresh frozen tissue at 12 months (11.2% less in Q175) and 18 months (14.4% less in Q175), with the difference between WT and Q175 significant in both cases (*p* = .00048; *p* = .02081, respectively). No differences between WT and Q175 striatal volume were seen for the fresh frozen tissue at 2 months of age (*p* = .96348; Figure 7). Thus, the dehydrating effects of brain fixation and processing appear to have masked a noteworthy and significantly lesser striatal volume in the native brain in the older Q175 mice, since without the dehydrating effects of brain fixation and processing, the volume reduction in Q175 mice compared to WT mice was 2–3 times greater in the fresh‐frozen than fixed tissue. Moreover, our observation that WT tissue shrank more during fixation than Q175 tissue shrank suggests a lesser water content in Q175 striatum at 6, 12, and 18 months of age than in WT striatum.

#### 3.2.13 Striatal interneurons

**FIGURE 8:**
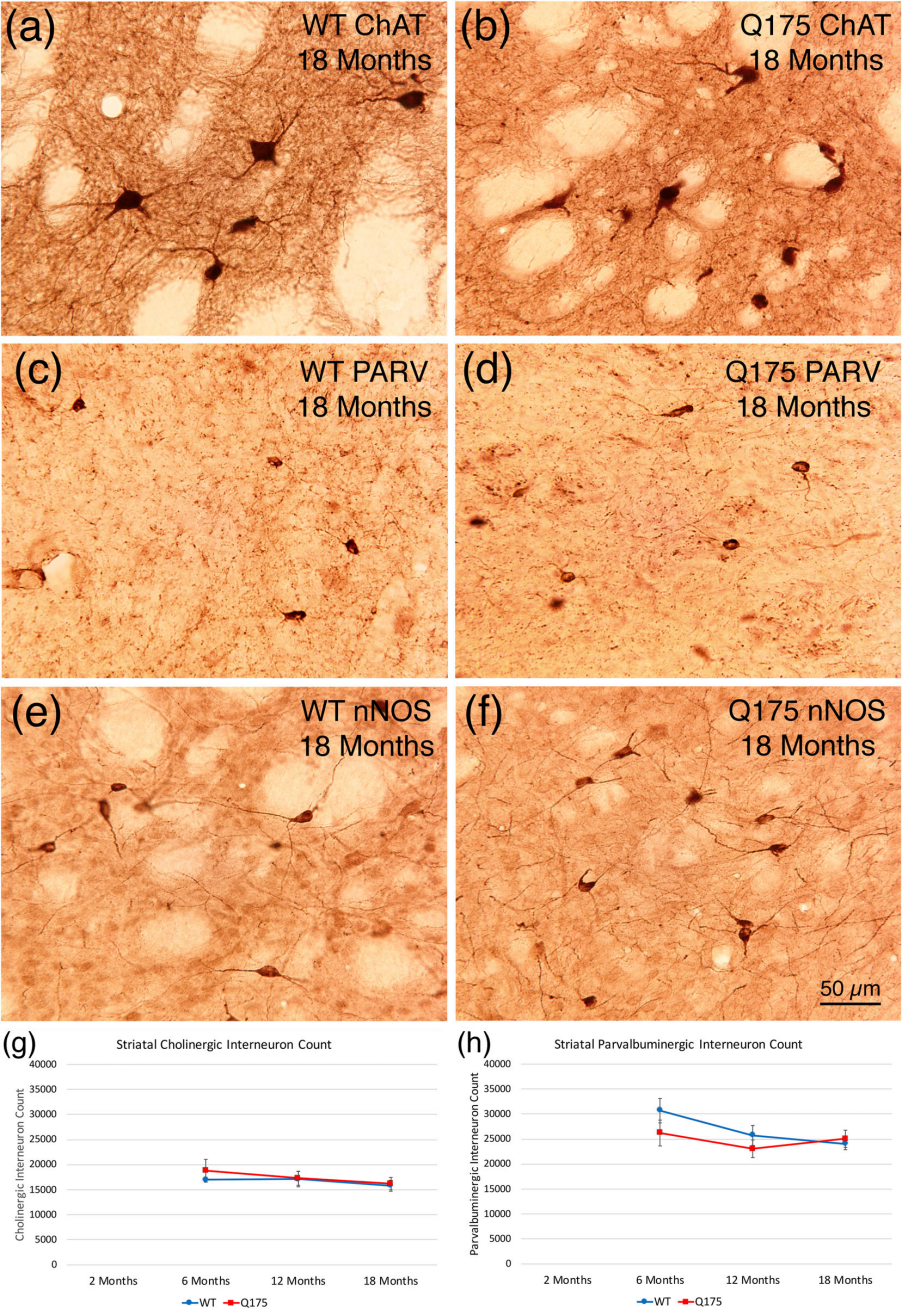
Images of striatal interneurons in WT and Q175 mice at 18 months of age (a–f), and graphs showing stereological counts of cholinergic (ChAT+) striatal interneurons (g) and of PARV+ striatal interneurons (h) in mutant and WT mice at 6, 12, and 18 months of age. Images (a) and (b) show striatal cholinergic interneurons in WT and Q175 mice at 18 months of age. Note that neuron abundance is comparable, but dendrites in Q175 mice seem shortened. Images (c) and (d) show striatal PARV+ interneurons in WT and Q175 mice at 18 months of age. Abundance and morphology do not obviously differ between mutant and WT. Images (e) and (f) show striatal nNOS+ interneurons in WT and Q175 mice at 18 months of age. Abundance and morphology do not obviously differ between mutant and WT. The scale bar in (f) pertains to all striatal interneuron images. Stereological counts of cholinergic (g) and PARV+ (h) striatal interneurons show that no significant difference is present in their abundance between WT and Q175 at 6, 12, or 18 months of age. Counts for nNOS+ striatal interneurons at 18 months also showed no difference between WT and Q175, and as a result this interneuron type was not assessed at earlier ages. Animal numbers for ChAT interneuron count: 6m WT *n* = 5, 6m Q175 *n* = 5; 12m WT *n* = 10, 12m Q175 *n* = 9; 18m WT *n* = 11, 18m Q175 *n* = 9. Animal numbers for PARV interneuron count: 6m WT *n* = 9, 6m Q175 *n* = 10; 12m WT *n* = 10, 12m Q175 *n* = 9; 18m WT *n* = 11, 18m Q175 *n* = 9. Animal numbers for nNOS interneuron count: 18m WT *n* = 11, 18m Q175 *n* = 9

**FIGURE 9:**
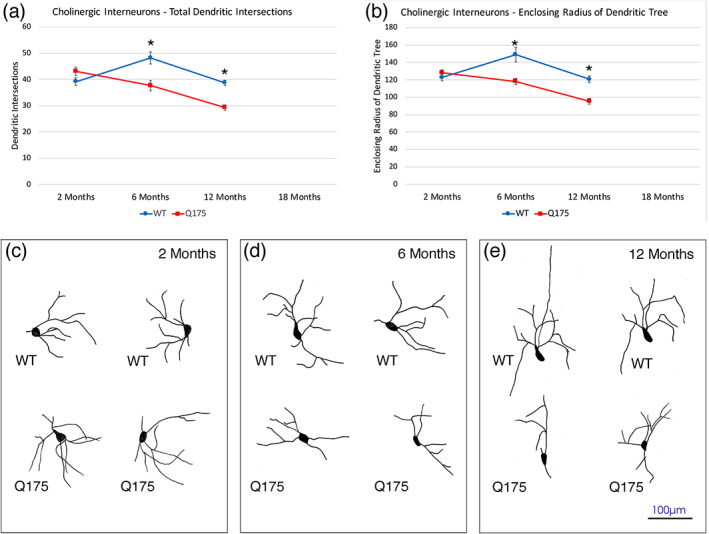
Graphs showing dendrite intersections as a function of distance from the soma (a) and enclosing radius of dendritic tree (b) in WT and mutant mice based on Sholl analysis, at 2, 6, and 12 months of age, and representative camera lucida drawings of ChAT+ cholinergic interneurons at high power in the striatum of WT and Q175 mice at 2 month (c), 6 months (d), and 12 months (e) of age. The results show that dendritic arborizations of ChAT+ interneurons were significantly decreased (asterisks) in Q175 heterozygous mice at 6 and 12 months of age. Scale bar in (e) applies to (c), (d), and (e). Animal numbers for ChAT interneuron Sholl analysis: 2m WT *n* = 10, 2m Q175 *n* = 10; 6m WT *n* = 10, 6m Q175 *n* = 10; 12m WT *n* = 10, 12m Q175 *n* = 9

We evaluated the abundance of the three major types of striatal interneurons in Q175 mice—the cholinergic tonically active neurons (TANs) immunolabeled for ChAT, the fast‐spiking interneurons (FSI) immunolabeled for PARV, and the low‐threshold spiking (LTS) interneurons immunolabeled for nNOS. We found no difference between mutant and WT mice at 6, 12, or 18 months in the abundance of cholinergic or PARV interneurons (Figure 8; Table 3). Because there was no loss for these at 6–18 months, we did not perform counts for cholinergic or PARV interneurons in our 2‐month‐old mice. Moreover, because there was no loss in nNOS+ striatal interneurons at 18 months (Table 3), we did not perform nNOS immunolabeling for the LTS striatal interneurons in 2‐, 6‐, or 12‐month‐old mice. In a prior study on male heterozygous Q140 mice, we used Sholl analysis to show that the dendrites of striatal cholinergic interneurons were significantly fewer and shorter in Q140 males at 1 and 4 months of age, although there was no reduction in cholinergic neuron abundance (Deng & Reiner, 2016). Because dendrite attenuation can affect cholinergic interneuron function, and since it might also occur in Q175 mice despite the absence of cholinergic interneuron loss, we conducted Sholl analysis on the immunolabeled 2‐, 6‐, and 12‐month‐old WT and Q175 cases (Figure 9). The analysis showed that the dendrites of striatal cholinergic interneurons were significantly fewer and shorter in heterozygous Q175 mice than in WT mice at 6 months (abundance, *p* = .00288; length, *p* = .00298) and 12 months of age (abundance, *p* = .0000005; length, *p* = .00012), but not at 2 months of age (abundance, *p* = .09229; length, *p* = .28662). Thus, despite the preservation in numbers, defects in the functioning of cholinergic interneurons are likely to be present in 6‐ and 12‐month‐old Q175 mice. Examination of striatal cholinergic interneurons in 18‐month‐old WT and Q175 mice suggests a similar dendritic reduction in mutant mice at this age.

**TABLE 3:** Results for the stereological counts for the three types of striatal interneurons examined in the present study, comparing WT and mutant at 6, 12, and 18 months

	Age in months	WT count	WT SEM	Q175 count	Q175 SEM	WT # of mice	Q175 # of mice	*p* value
ChAT count	2	Not done	Not done	Not done	Not done	Not done	Not done	Not done
ChAT count	6	16,979.3	±467.5	18,799.5	±2,242.5	5	5	.44977
ChAT count	12	17,134.3	±1,551.4	17,263.5	±1,375.5	10	9	.95148
ChAT count	18	15,787.7	±1,087.9	16,210.3	±1,230.4	11	9	.79939
PARV count	2	Not done	Not done	Not done	Not done	Not done	Not done	Not done
PARV count	6	30,683.5	±2,439.7	26,208.5	±2,583.9	9	10	.27126
PARV count	12	25,722.5	±1,999.3	23,065.4	±1,757.8	10	9	.34163
PARV count	18	24,013.4	±1,145.6	25,051.1	±1,719.9	11	9	.57139
nNOS count	2	Not done	Not done	Not done	Not done	Not done	Not done	Not done
nNOS count	6	Not done	Not done	Not done	Not done	Not done	Not done	Not done
nNOS count	12	Not done	Not done	Not done	Not done	Not done	Not done	Not done
nNOS count	18	25,279.3	±1,516.2	26,745.4	±964.3	11	9	.44905

*Note*: Counts are presented as the mean ± *SEM*. Unpaired two‐tailed t‐tests were used to assess the statistical significance of any differences between WT and Q175 mice at each age. We did not perform immunolabeling for nNOS+ at 6 or 12 months because there was no loss in nNOS+ neurons at 18 months. Additionally, counts were not performed at 2 months for any of the interneuron types, because of the absence of differences at any of the older ages.

### 3.3 Neurons in striatal target areas

#### 3.3.1 GPe—Protoypical PARV neurons

**FIGURE 10:**
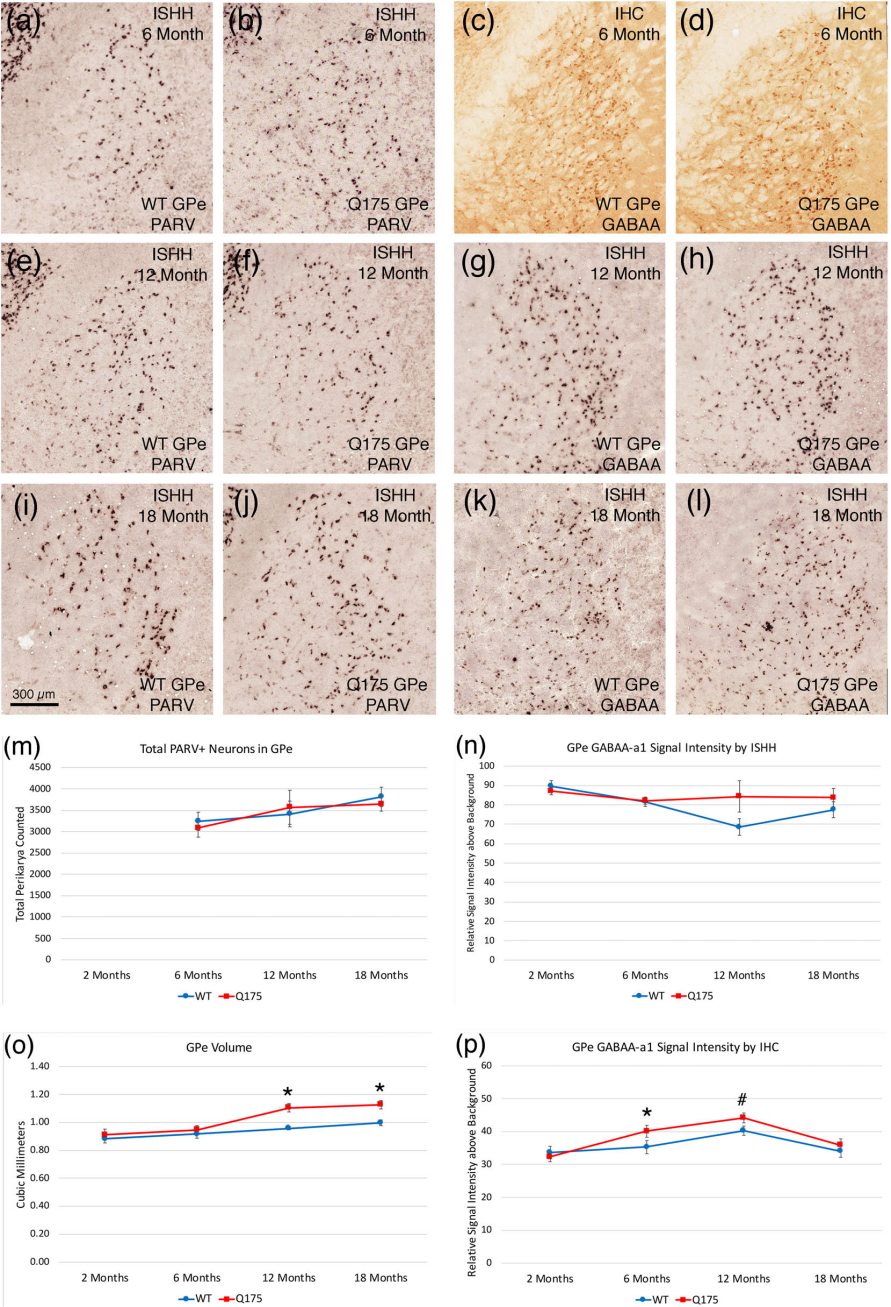
Images showing PARV ISHH in GPe of WT and Q175 mice at 6, 12, and 18 months (a, b, e, f, i, j), images showing GABAA‐α1 immunolabeling in GPe of WT and Q175 mice at 6 months (c, d), images showing GABAA‐α1 ISHH in GPe of WT and Q175 mice at 12 and 18 months (g, h, k, l), graph showing total PARV neuron abundance in GPe in WT and Q175 mice at 6, 12, and 18 months (m), graph showing ISHH GABAA‐α1 signal intensity for GPe neurons in WT and Q175 mice at 2, 6, 12, and 18 months (n), graph showing GPe volume in WT and Q175 mice at 2, 6, 12, and 18 months (o), and graph showing IHC GABAA‐α1 signal intensity for GPe neurons in WT and Q175 at 2, 6, 12, and 18 months (p). The results show that PARV neuron abundance in GPe does not differ between WT and mutants as mice age, but GPe volume is significantly greater than in sham at 12 and 18 months (asterisks). GABAA‐α1 signal was significantly elevated in Q175 mice at 6 months in immunolabeled tissue (asterisk), and also trended toward elevation at 12 months in the immunolabeled tissue (pound symbol). A trend toward elevation in signal in mutants was also seen in the ISHH tissue at 12 months. Scale bar in image (i) applies to all images. All images are from sections in the coronal plane, with dorsal to the top and medial to the left. Animal numbers for PARV count in GPe count: 6m WT *n* = 10, 6m Q175 *n* = 10; 12m WT *n* = 10, 12m Q175 *n* = 9; 18m WT *n* = 6, 18m Q175 *n* = 6. Animal numbers for GPe volume: 2m WT *n* = 10, 2m Q175 *n* = 10; 6m WT *n* = 10, 6m Q175 *n* = 10; 12m WT *n* = 10, 12m Q175 *n* = 9; 18m WT *n* = 11, 18m Q175 *n* = 9. Animal numbers for ISHH GABAA‐α1 neuronal signal in GPe: 2m WT *n* = 10, 2m Q175 *n* = 10; 6m WT *n* = 10, 6m Q175 *n* = 10; 12m WT *n* = 9, 12m Q175 *n* = 10; 18m WT *n* = 10, 18m Q175 *n* = 10. Animal numbers for IHC GABAA‐α1 neuronal signal in GPe: 2m WT *n* = 10, 2m Q175 *n* = 10; 6m WT *n* = 10, 6m Q175 *n* = 10; 12m WT *n* = 10, 12m Q175 *n* = 10; 18m WT *n* = 4, 18m Q175 *n* = 6

No abnormalities were evident in the Q175 mutants in the total abundance of PARV+ prototypical neuron in GPe at 6, 12, or 18 months, as determined from the immunolabeled tissue (Figure 10). ISHH also found no difference between Q175 mice and WT mice in the spatial density of PARV+ neurons in GPe or in the intensity of PARV signal per neuron at 6, 12, and 18 months. Because PARV neuron abundance and labeling intensity were normal in Q175 mice compared to WT at 6 months, they were not assessed at 2 months. The image analysis of the ENK‐immunostained material did reveal, however, that the volume of GPe was significantly greater in Q175 mice than in WT mice at 12 months (*p* = .00020) and 18 months (*p* = .00252) but not at 2 and 6 months. This increased volume is likely to be associated with the increased iSPN fiber abundance in GPe.

#### 3.3.2 GPe—Arkypallidal neurons

**FIGURE 11:**
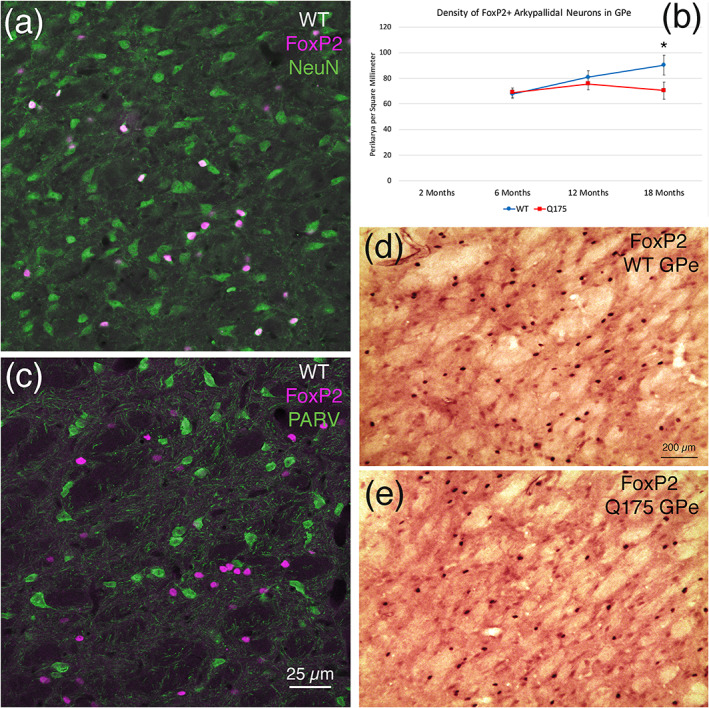
CLSM image showing immunofluorescence double‐label for NeuN (green) and FoxP2 (magenta) in WT GPe (a), graph showing the spatial density of FoxP2+ arkypallidal neurons in GPe in WT and Q175 mice at 6, 12, and 18 months of age (b), CLSM image showing immunofluorescence double‐label for PARV (green) and FoxP2 (magenta) in WT GPe (c), and images showing PAP‐immunolabeling for FoxP2 in GPe of WT and Q175 mice at 18 months of age (d, e). The results show that FoxP2 in GPe is localized to neurons (a), but not PARV+ prototypical neurons (c), and that arkypallidal neurons are reduced at 12 and 18 months in Q175 mice, significantly so at 18 months (b) (asterisk). All images are from sections in the coronal plane, with dorsal to the top and medial to the left. Animal numbers for FoxP2 IHC in GPe: *n* = 8; 6m WT, 6m Q175 *n* = 9; 12m WT *n* = 10, 12m Q175 *n* = 9; 18m WT *n* = 8, 18m Q175 *n* = 9

Because several studies have shown that the arkypallidal neurons of GPe can be identified by their expression of FoxP2 (Abdi et al., 2015; Hernández et al., 2015), we used immunolabeling for FoxP2 to identify arkypallidal neurons in WT and Q175 mice. To establish that FoxP2 immunolabeling in GPe occurred only in neurons and was not found in prototypical PARV+ neurons of GPe, we used multiple immunofluorescence labeling of WT tissue double‐labeled for NeuN and FoxP2, or PARV and FoxP2. CLSM viewing of the multiple‐labeled tissue confirmed that FoxP2 in GPe only occurred in neurons (as detected with anti‐NeuN), and was not present in PARV+ neurons (Figure 11a,c). Having thereby confirmed FoxP2 as a marker for non‐prototypical presumptive arkypallidal neurons in GPe, we used FoxP2‐immunolabeling to assess the spatial density of arkypallidal neurons in GPe at 6, 12, and 18 months of age in WT and Q175 mice. We saw no reduction in the spatial density of arkypallidal neurons in GPe at 6 months of age (*p* = .41723) in Q175 mice (Figure 11b). We also saw no significant reduction in the spatial density of FoxP2+ arkypallidal neurons in GPe at 12 months of age in the Q175 mice, although mutants had 6.6% fewer (*p* = .22325; Figure 11b). We did, however, see a significant 22.0% reduction (*p* = .03403) in the spatial density of FoxP2+ arkypallidal neurons in GPe at 18 months of age in the Q175 mice (Figure 11b,d,e). For these studies comparing across ages, we used the fresh‐frozen tissue due to limitations on the availability of fixed tissue at 6 and 12 months of age. To further evaluate the loss of arkypallidal neurons at 18 months, we quantified the abundance of FoxP2 neurons relative to all neurons in GPe (immunolabeled for NeuN), using a subset of the fixed tissue from WT and mutant cases that was available for this age. We found that the abundance of arkypallidal neurons as a percent of NeuN+ neurons in GPe was significantly less (*p* = .03094) in Q175 mice (33.4% less) at 18 months than in WT mice of the same age. Thus, arkypallidal GPe neurons appear to sustain significant 20–30% loss by 18 months of age.

#### 3.3.3 GPe neurons—GABAA‐α1

Because loss or hypoactivity of the striatal input to its target areas is associated with an upregulation of GABAA receptors, especially the α1 subunit, we assessed localization of this receptor subunit in GPe by immunolabeling and by ISHH. As shown in Figure 10, we observed no significant difference between WT and Q175 mice in GPe neuron labeling intensity or in the spatial density of neurons labeled for GABAA‐α1 at 2, 6, 12, or 18 months of age by ISHH, although a slight trend toward increased signal intensity was seen at 12 months. We also found that there was no significant difference between WT and Q175 mice in the total abundance of GPe neurons immunolabeled for the GABAA‐α1 subunit mice at 2, 6, or 12 months (Figure 10), but there was a slight but significant increase in the immunolabeling intensity of GPe neurons for GABAA‐α1 in Q175 mice at 6 months (*p* = .04741), and a trend toward an increase at 12 months (*p* = .06900). The increase seen with immunolabeling was no longer evident at 18 months, although a slight increase in the total abundance of GABAA‐α1 neurons in mutant mice that trended toward statistical significance (*p* = .05318) was seen at 18 months. Thus, the GABAA‐α1 data showed only a weak tendency to be increased in the mutant mice as they aged. Nonetheless, a correlation with rotarod performance was seen—rotarod performance was worse with more GABAA‐α1 neurons in GPe at 18 months, with a significant −0.685 correlation between GABAA‐α1 neuron abundance in GPe and rotarod latency to fall. This suggests a possible role of GPe dysfunction as reflected in an increased GPe expression of GABAA‐α1 in motor impairment at this age.

#### 3.3.4 GPi—PARV neurons

**FIGURE 12:**
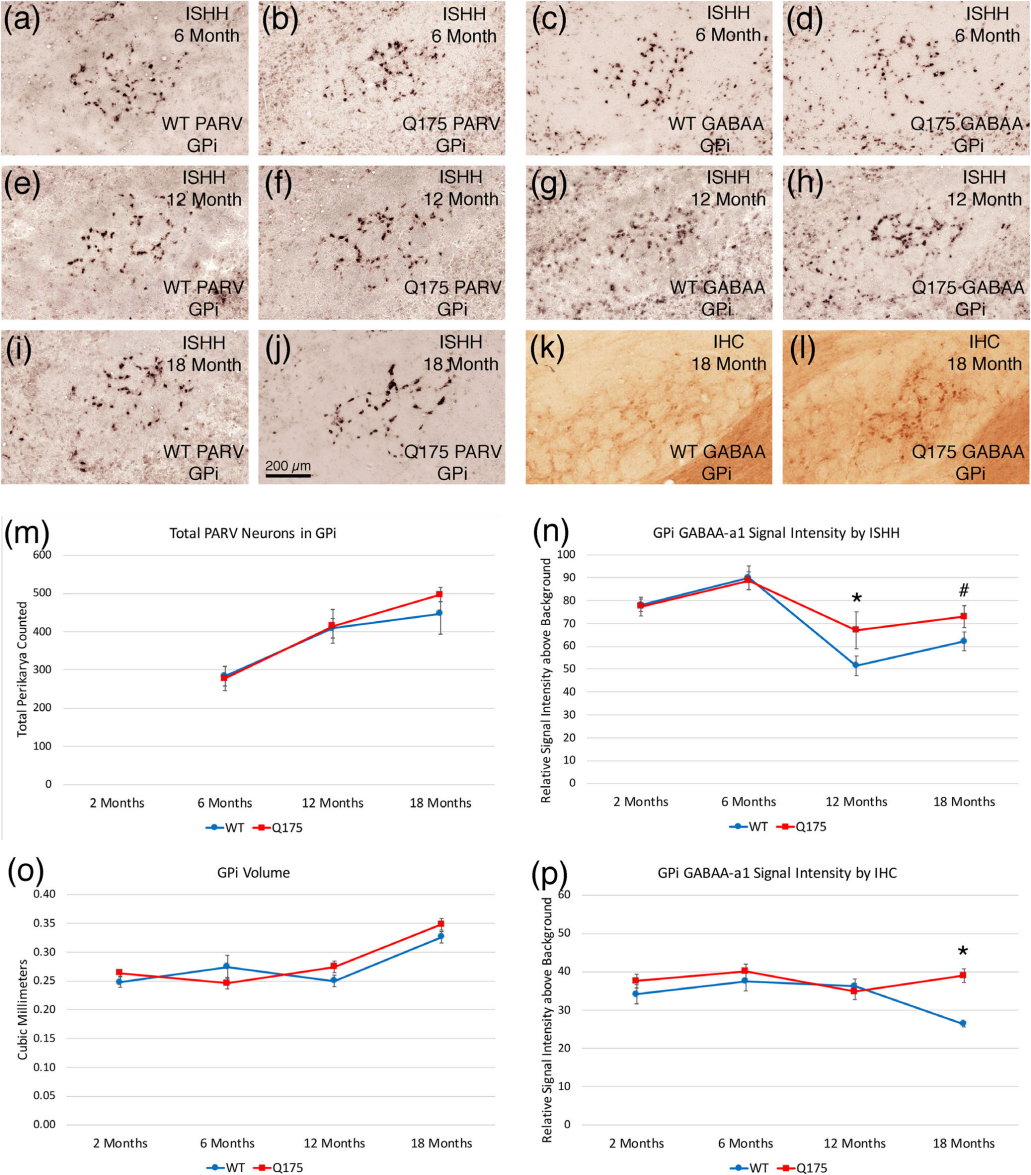
Images showing PARV ISHH in GPi of WT and Q175 mice at 6, 12, and 18 months (a, b, e, f, i, j), images showing GABAA‐α1 ISHH in GPi of WT and Q175 mice at 6 and 12 months (c, d, g, h), images showing GABAA‐α1 IHC in GPi of WT and Q175 mice at 18 months (k, l), graph showing total PARV neuron abundance in GPi in WT and Q175 mice at 6, 12, and 18 months (m), graph showing ISHH GABAA‐α1 signal intensity for GPi neurons in WT and Q175 mice at 2, 6, 12, and 18 months (n), graph showing GPi volume in WT and Q175 mice at 2, 6, 12, and 18 months (o), and graph showing IHC GABAA‐α1 signal intensity for GPi neurons in WT and Q175 at 2, 6, 12, and 18 months (p). The results show that neither PARV neuron abundance in GPi nor GPi volume differed between WT and mutants as mice age, although GPi volume trended toward greater than sham at 12 and 18 months. GABAA‐α1 signal was elevated in Q175 at 12 months in ISHH tissue (asterisk), and trended toward elevation at 18 months in the ISHH tissue (pound symbol), and was significantly elevated in the IHC tissue at 18 months (asterisk). Scale bar in image (j) applies to all images. All images are from sections in the coronal plane, with dorsal to the top and medial to the left. Animal numbers for PARV count in GPi count: 6m WT *n* = 10, 6m Q175 *n* = 10; 12m WT *n* = 10, 12m Q175 *n* = 9; 18m WT *n* = 11, 18m Q175 *n* = 9. Animal numbers for GPi volume: 2m WT *n* = 10, 2m Q175 *n* = 10; 6m WT *n* = 10, 6m Q175 *n* = 10; 12m WT *n* = 10, 12m Q175 *n* = 9; 18m WT *n* = 11, 18m Q175 *n* = 9. Animal numbers for ISHH GABAA‐α1 neuronal signal in GPi: 2m WT *n* = 10, 2m Q175 *n* = 10; 6m WT *n* = 10, 6m Q175 *n* = 10; 12m WT *n* = 10, 12m Q175 *n* = 10; 18m WT *n* = 10, 18m Q175 *n* = 10. Animal numbers for IHC GABAA‐α1 neuronal signal in GPi: 2m WT *n* = 10, 2m Q175 *n* = 10; 6m WT *n* = 10, 6m Q175 *n* = 10; 12m WT *n* = 10, 12m Q175 *n* = 10; 18m WT *n* = 4, 18m Q175 *n* = 6

No significant neuronal pathology was evident in GPi as assessed using PARV ISHH or PARV immunolabeling at 6, 12, and 18 months of age. Neuronal abundance for the entire GPi as assessed by immunolabeling or the spatial density of PARV neurons in GPi as assessed by ISHH did not differ significantly between mutants and WT mice, nor did PARV labeling intensity (Figure 12). Because PARV neuron abundance and labeling intensity were normal at 6 months, they were not assessed at 2 months. Additionally, the volume of GPi did not differ between mutant and WT mice at 2–18 months, based on the image analysis of the SP‐immunostained material (Figure 12).

#### 3.3.5 GPi neurons—GABAA‐α1

Based on analysis of the ISHH tissue labeled for GABAA‐α1 and the tissue immunolabeled for GABAA‐α1, there were no significant differences between mutant and WT in the intensity of GABAA‐α1 signal for GPi neurons at 2 or 6 months of age, although we did see a trend toward an increased spatial density of GPi neurons labeled by ISHH for GABAA‐α1 at 6 months (*p* = .08864; Figure 12). In 12‐month‐old mice, there was a significant increase in the GABAA‐α1 neuronal signal for GPi neurons by ISHH (*p* = .03488), suggesting hypofunction in the striato‐GPi circuit by this age. Similarly, immunolabeling also revealed a highly significant increase in the GABAA‐α1 signal in GPi neurons at 18 months (*p* = .00057), consistent with our SP immunolabeling data showing an abnormality in SP+ terminals in GPi at 18 months (Figure 12), and ISHH showed a trend in the same direction for GABAA‐α1 signal in GPi neurons (*p* = .12720). The GABAA‐α1 data showed correlations with the behavioral data for these mice, further reinforcing the possible role of the circuit‐level abnormalities revealed by the GABAA‐α1 localization in GPi in motor dysfunction. For example, the elevated GABAA‐α1 signal intensity in the GPi of 18‐month‐old Q175 mice was significantly inversely associated with turn radius (−0.779), meaning the turning ability diminished as GABAA‐α1 signal on GPi neurons increased. Thus, the motor impairment reflected in the narrower turning angles was associated with the striato‐GPi hypofunction implied by the elevated GABAA‐α1 signal on GPi neurons.

#### 3.3.6 SNr—PARV neurons

**FIGURE 13:**
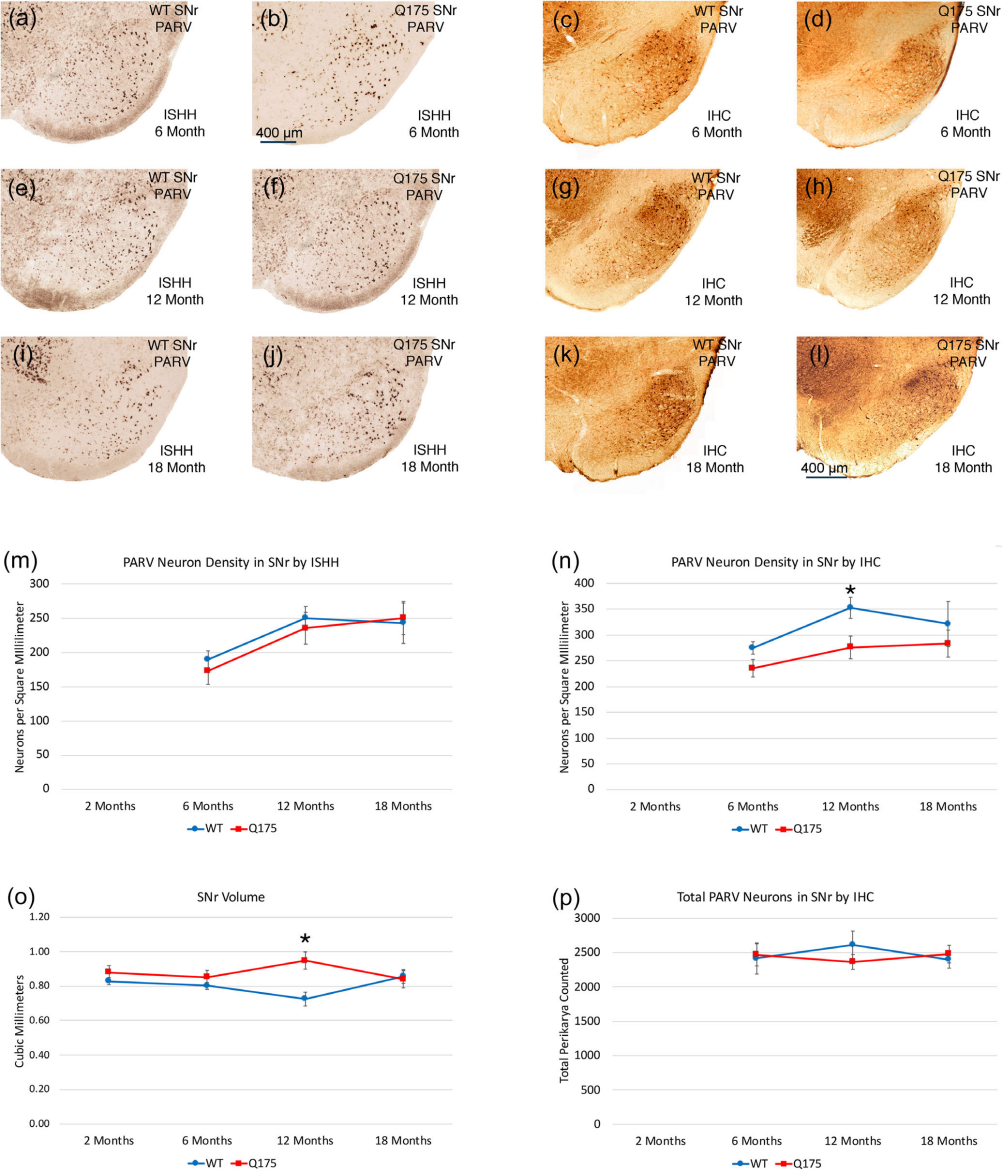
Images showing PARV ISHH in SNr of WT and Q175 mice at 6, 12, and 18 months (a, b, e, f, i, j), images showing PARV IHC in SNr of WT and Q175 mice at 6, 12, and 18 months (c, d, g, h, k, l), graph showing total PARV neuron density in SNr by ISHH in WT and Q175 mice at 6, 12, and 18 months (m), graph showing PARV neuron density in SNr by IHC in WT and Q175 mice at 2, 6, 12, and 18 months (n), graph showing SNr volume in WT and Q175 mice at 2, 6, 12, and 18 months (o), and graph showing total PARV neuron count in SNr by IHC in WT and Q175 at 6, 12, and 18 months (p). The results show that PARV neuron density in SNr as determined by ISHH did not differ between WT and mutants as mice age, although it was less in mutants at 12 months by IHC, the same age at which the volume of SNr was significantly increased as well. Overall neuron abundance in SNr did not differ significantly between mutant and WT at any age. The scale bar in (b) pertains to all ISHH images, while that in image (l) pertains to all immunolabeling images. All images are from sections in the coronal plane, with dorsal to the top and medial to the left. Animal numbers for PARV density in SNr by ISHH: 6m WT *n* = 9, 6m Q175 *n* = 10; 12m WT *n* = 10, 12m Q175 *n* = 10; 18m WT *n* = 10, 18m Q175 *n* = 10. Animal numbers for SNr volume: 2m WT *n* = 10, 2m Q175 *n* = 10; 6m WT *n* = 10, 6m Q175 *n* = 10; 12m WT *n* = 10, 12m Q175 *n* = 9; 18m WT *n* = 11, 18m Q175 *n* = 8. Animal numbers for PARV density in SNr by IHC: 2m WT *n* = 10, 2m Q175 *n* = 10; 6m WT *n* = 10, 6m Q175 *n* = 9; 12m WT *n* = 10, 12m Q175 *n* = 9; 18m WT *n* = 9, 18m Q175 *n* = 9. Animal numbers for total PARV count in SNr by IHC: 6m WT *n* = 10, 6m Q175 *n* = 10; 12m WT *n* = 10, 12m Q175 *n* = 8; 18m WT *n* = 15, 18m Q175 *n* = 13

No significant change in overall PARV+ neuron abundance was detected at 6 months of age (Figure 13) in counts of PARV neurons in SNr in the immunostained material (102.2% of WT), or for PARV+ neuron spatial density in SNr assessed by ISHH (91.2%). ISHH labeling intensity of individual PARV+ neurons in SNr at 6 months was also no different in Q175 mice than in WT mice. ISHH also did not detect a difference between mutant and WT mice in the spatial density of PARV+ SNr neurons at 12 or 18 months (Figure 13). Immunolabeling, however, detected a generally higher spatial density of PARV+ neurons in WT than mutant mice at 6, 12, and 18 months of age, with a significant difference, observed at 12 months (*p* = .02009; Figure 13). This difference in spatial density does not appear to stem from PARV+ neuron loss, since mutant and WT mice did not differ significantly in total PARV+ SNr neurons at 6, 12, or 18 months of age (Figure 13). Note that, based on the image analysis of the SP‐immunostained material, the volume of SNr was consistently greater in mutant mice across the ages examined, and was significantly more at 12 months in the mutants than in WT mice (*p* = .000001; 130.8% of WT). Thus, the generally lower spatial density of PARV+ SNr neurons in mutants may reflect the generally greater SNr volume in the mutants, rather than PARV+ neuron loss, particularly at 12 months.

#### 3.3.7 SNr neurons—GABAA‐α1

**FIGURE 14:**
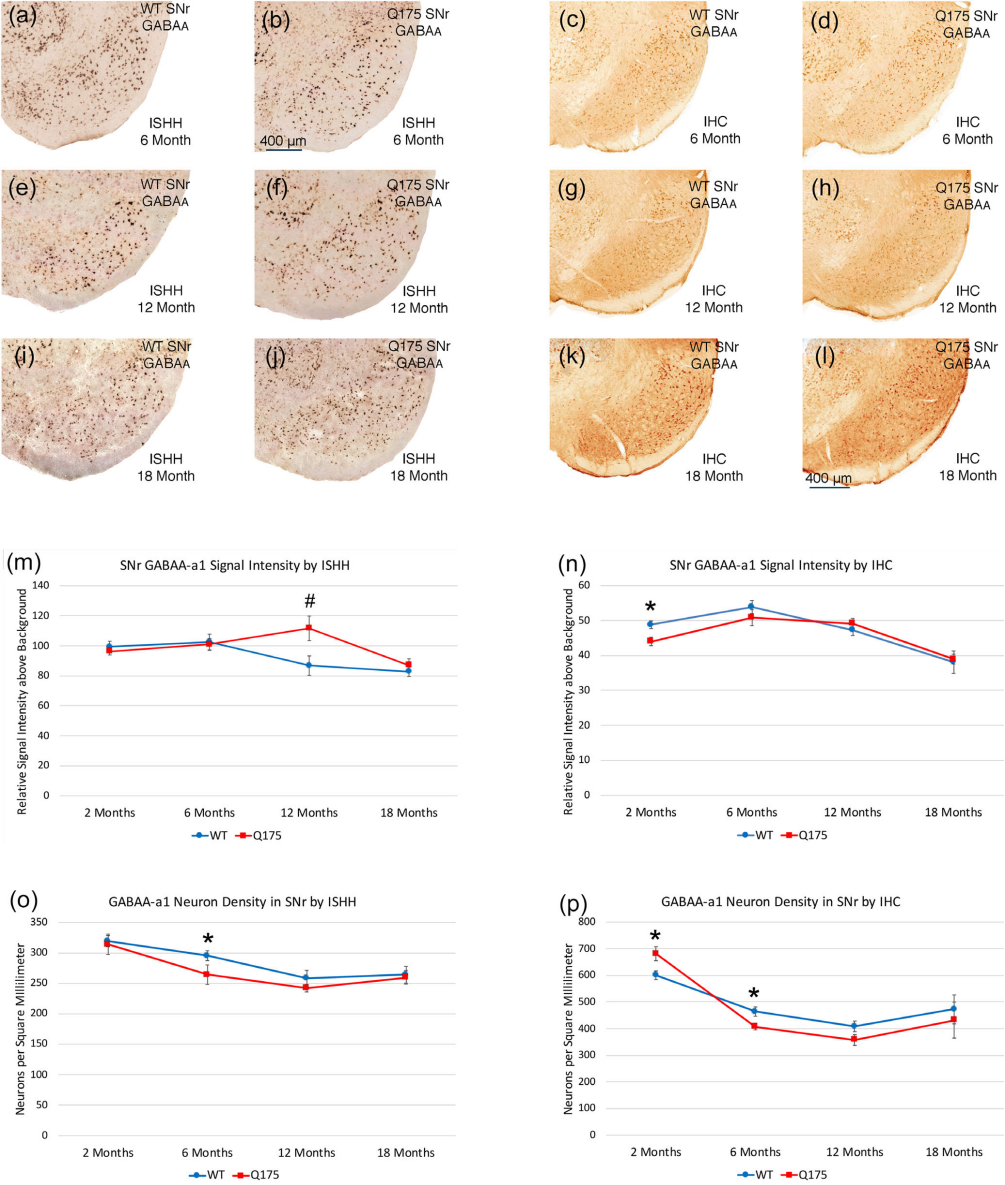
Images showing GABAA‐α1 ISHH in SNr of WT and Q175 mice at 6, 12, and 18 months (a, b, e, f, i, j), images showing GABAA‐α1 IHC in SNr of WT and Q175 mice at 6, 12, and 18 months (c, d, g, h, k, l), graph showing total GABAA‐α1 neuronal intensity in SNr by ISHH in WT and Q175 mice at 2, 6, 12, and 18 months (m), graph showing GABAA‐α1 neuronal intensity in SNr by IHC in WT and Q175 mice at 2, 6, 12, and 18 months (n), graph showing GABAA‐α1 neuronal density in SNr by ISHH in WT and Q175 mice at 2, 6, 12, and 18 months (o), and graph showing GABAA‐α1 neuronal density in SNr by IHC in WT and Q175 at 2, 6, 12, and 18 months (p). The results show that GABAA‐α1 neuronal intensity and density in SNr as determined by ISHH did not differ notably or consistently between WT and mutants as mice age, although a slight trend toward increased signal in mutants was seen at 12 months. In contrast, intensity was less in mutants, but density was more in mutants at 2 months by IHC. By 6 months, however, density was slightly less in mutants by IHC, as well as by ISHH. The scale bar in (b) pertains to all ISHH images, while that in image (l) pertains to all immunolabeling images. All images are from sections in the coronal plane, with dorsal to the top and medial to the left. Animal numbers for GABAA‐α1 intensity in SNr by ISHH: 2m WT *n* = 10, 2m Q175 *n* = 10; 6m WT *n* = 10, 6m Q175 *n* = 9; 12m WT *n* = 10, 12m Q175 *n* = 10; 18m WT *n* = 10, 18m Q175 *n* = 10. Animal numbers for GABAA‐α1 neuronal density in SNr by ISHH: 2m WT *n* = 10, 2m Q175 *n* = 10; 6m WT *n* = 10, 6m Q175 *n* = 9; 12m WT *n* = 10, 12m Q175 *n* = 10; 18m WT *n* = 10, 18m Q175 *n* = 10. Animal numbers for GABAA‐α1 intensity in SNr by IHC: 2m WT *n* = 10, 2m Q175 *n* = 10; 6m WT *n* = 6, 6m Q175 *n* = 5; 12m WT *n* = 10, 12m Q175 *n* = 10; 18m WT *n* = 4, 18m Q175 *n* = 6. Animal numbers for GABAA‐α1 neuronal density in SNr by IHC: 2m WT *n* = 10, 2m Q175 *n* = 10; 6m WT *n* = 6, 6m Q175 *n* = 5; 12m WT *n* = 10, 12m Q175 *n* = 10; 18m WT *n* = 4, 18m Q175 *n* = 6

Immunolabeling detected significantly lower GABAA‐α1 neuron labeling intensity (*p* = .00882) but significantly greater GABAA‐α1 neuron spatial density (*p* = .01925) in Q175 SNr than WT SNr at 2 months of age. No difference was seen, however, between WT and Q175 mice in GABAA‐α1 neuron labeling intensity or spatial density by ISHH at 2 months of age (Figure 14). ISHH also detected no significant differences in GABAA‐α1 signal intensity between mutant and WT at 6, 12, or 18 months, although Q175 GABAA‐α1 SNr neurons were significantly lower in spatial density than WT at 6 months of age in the ISHH tissue (*p* = .04573). Immunolabeling detected no differences between mutant and WT mice in intensity or abundance of GABAA‐α1 SNr neurons at 12 or 18 months, although like with ISHH, Q175 GABAA‐α1 SNr neurons were significantly lower in spatial density than WT at 6 months of age (*p* = .02277). The lower spatial density of GABAA‐α1 SNr neurons in mutants also may reflect the generally greater SNr volume in mutants.

#### 3.3.8 STN neurons

**FIGURE 15:**
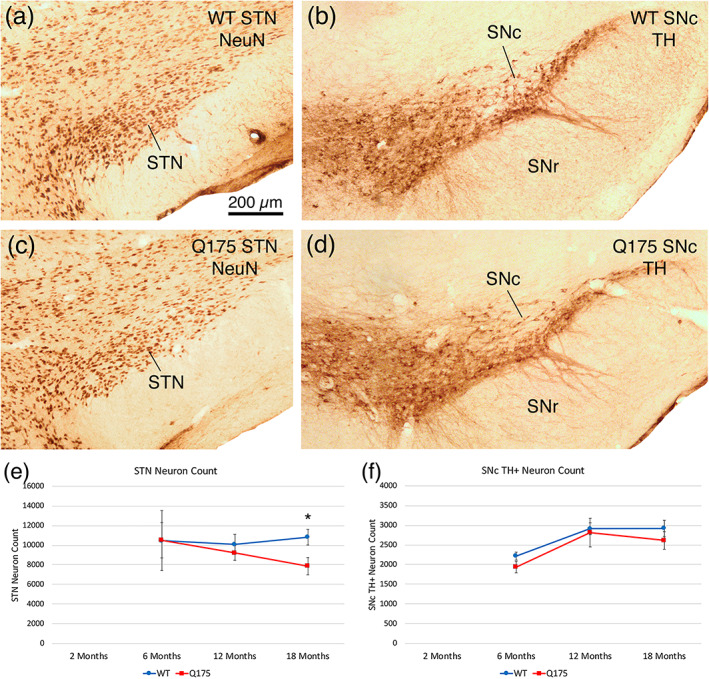
Image showing NeuN immunolabeling of subthalamic nucleus (STN) in WT mouse at 18 months of age (a), image showing tyrosine hydroxylase (TH) immunolabeling of substantia nigra pars compacta (SNc) in WT mouse at 18 months of age (b), image showing NeuN immunolabeling of STN in Q175 mouse at 18 months of age (c), image showing TH immunolabeling of SNc in Q175 mouse at 18 months of age (d), graph showing stereological counts of STN neurons in WT and Q175 mice at 6, 12, and 18 months of age (e), and graph showing counts of TH+ SNc neurons in WT and Q175 mice at 6, 12, and 18 months of age (f). As shown, significant STN neuron loss (asterisk) occurs by 18 months in mutants, but no loss is seen of TH+ neurons in SNc. Scale bar in image (a) applies to all images. All images are from sections in the coronal plane, with dorsal to the top and medial to the left. Animal numbers for STN count: 6m WT *n* = 5, 6m Q175 *n* = 5; 12m WT *n* = 10, 12m Q175 *n* = 9; 18m WT *n* = 11, 18m Q175 *n* = 9. Animal numbers for SNc count: 6m WT *n* = 10, 6m Q175 *n* = 10; 12m WT *n* = 10, 12m Q175 *n* = 9; 18m WT *n* = 11, 18m Q175 *n* = 8

We saw no significant loss of STN neurons in Q175 mice at 6 or 12 months of age, but we did find a significant 25.9% STN neuron loss in mutants at 18 months of age (*p* = .01799), consistent with the findings of Atherton et al. (2016) (Figure 15). Because no loss was seen at 6 months, 2 months was not examined.

#### 3.3.9 SNc neurons

Counts of TH+ SNc neurons showed no difference between mutant and WT mice at any of the three ages examined, 6, 12, and 18 months (Figure 15). Because no loss was seen at 6 months, 2 months was not examined.

#### 3.3.10 NIIs: Localization

**FIGURE 16:**
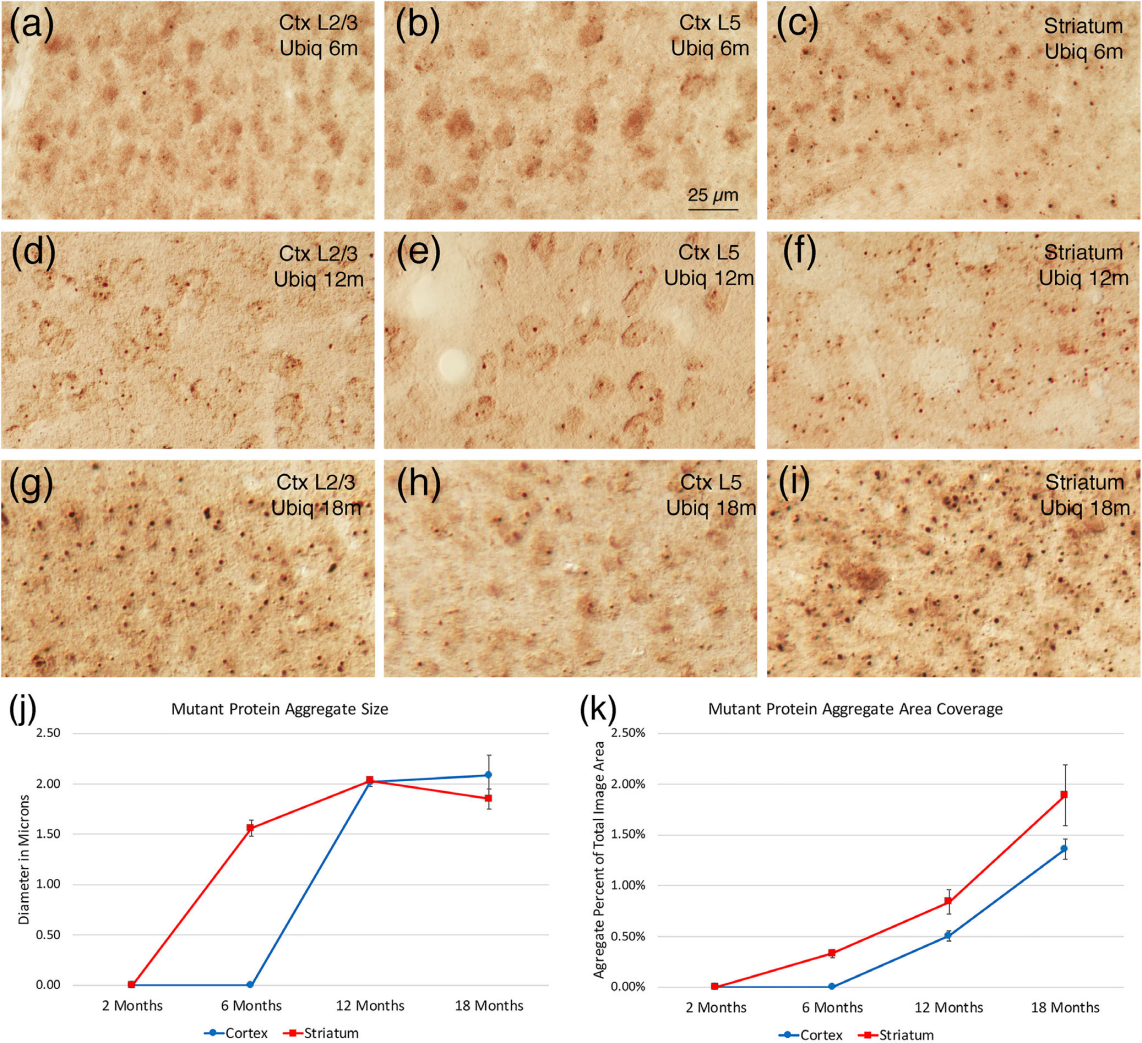
Images of cortical layers 2–3 (a, d, g), cortical Layer 5 (b, e, h), and striatum (c, f, i) from sections from a 6‐month‐old Q175 mouse (a, b, c), 12‐month‐old Q175 mouse (d, e, f), and 18‐month‐old Q175 mouse (g, h, i) that had been antigen retrieved and then immunostained with anti‐ubiquitin, and graphs quantifying NII size (j) and areal coverage (k) in cortex and striatum of Q175 mice at 2, 6, 12, and 18 months of age. Aggregates are negligible in cerebral cortex until 12 months of age but widespread (albeit small) in striatum already at 6 months of age. Magnification is the same for all images. Animal numbers for cortex and striatum: 2m Q175 *n* = 10, 6m Q175 *n* = 10; 12m Q175 *n* = 6; 18m Q175 *n* = 9

Anti‐ubiquitin immunolabeling to detect mutant protein aggregates revealed that neither striatum nor cortex contained aggregates in 2‐month‐old Q175 mice. In contrast, striatum contained perikaryal aggregates that appeared to be NIIs in Q175 mice at 6 months, with few neuropil aggregates. Cortical aggregates were rare, although small perikaryal aggregates were seen in Layer 4 neurons and in some Layer 2/3 neurons (Figure 16). Aggregates were sparse and small in GPe, GPi, and SNr at 6 months, and largely confined to the neuropil. The STN and SNc were largely devoid of aggregates. Using ImageJ to measure striatal aggregates in 6‐month‐old Q175 mice (Figure 16), we confirmed that striatal aggregates were relatively small yet (about 1.5 μm in diameter), and occupied only a small fraction of the striatal area (0.33%). At 12 months, aggregates were much more abundant in cortex and striatum, and appeared to be predominantly nuclear. In cortex, all Layer 4 neurons possessed aggregates, and aggregates were now common in Layers 2/3 as well, and typically larger than in Layer 4. Layer 6 neurons also typically possessed aggregates, as did Layer 5A neurons, but aggregates were sparser in Layer 5B neurons. Neuropil aggregates were more common and larger in GPe, GPi, and SNr than at 6 months, but aggregates remained sparse in SNc and STN. ImageJ showed that aggregates at 12 months of age were about 2 μm in size (Figure 16), and were abundant in upper layers of cerebral cortex and in striatum, with NIIs predominant. They were less abundant spatially in cortical layers 5–6, where neurons are larger and less tightly packed. Overall aggregates occupied 0.84% of striatum and 0.51% of cortex at 12 months. At 18 months, both cerebral cortex and striatum were highly enriched in cellular aggregates that appeared to be NIIs, and striatum was additionally now enriched in neuropil aggregates in Q175 mice (Figure 16). In cortex, aggregates were widespread in all layers, except for Layer 5B. In GPe, GPi, and SNr at 18 months, punctate labeling was now relatively dense and likely to represent mutant protein aggregates in SPN terminals, given that SPNs transport huntingtin to their terminals (Reiner & Deng, 2018). Aggregates remained sparse in SNc and STN, although some neuropil aggregates were evident. Based on ImageJ measurements of aggregates in cortex and striatum at 18 months (Figure 16), the aggregates were again about 2 μm in size, and occupied 1.36% of cortical area and 1.89% of striatal area. Because of the emergence of neuropil aggregates in striatum, which were smaller than the perikaryal aggregates, mean aggregate size was somewhat less than at 12 months, although the areal coverage by aggregated mutant protein was greater.

#### 3.3.11 NIIs—Correlation with neuropathology

Among 6‐month‐old Q175 mice, the percent of the striatum covered by aggregates tended to inversely correlate with the abundance of ENK‐immunostained GPe fibers (−0.481) and with the abundance of SP‐immunostained SN fibers (−0.540). Although this was short of significance for an *n* = 10, this provides suggestive evidence that the abnormally increased abundance of ENK‐immunostained GPe fibers and SP‐immunostained SN fibers were less great in Q175 mice with greater aggregate burden, suggesting a protective effect of SPN aggregates for this SPN terminal abnormality. Similarly, at 12 months, the percent of the striatum covered by aggregates tended to inversely correlate with the abundance of SP‐immunostained GPi fibers (−0.793), as did the percent of the cortex covered by aggregates (−0.722). The abundance of DARPP32+ striatal neurons showed a more complicated relationship to aggregate load. Among 6‐month‐old Q175 mice, the aggregate abundance was associated with a harmful effect, as the percent of the striatum covered by aggregates tended to inversely correlate with the abundance of DARPP32+ striatal neurons (−0.595). In 18‐month‐old Q175 mice, however, DARPP32+ neuron abundance was highly correlated with striatal aggregate load (*r* = .616), suggesting that mutant protein aggregation protected this neurochemical feature of SPNs at this age. It may be that mutant protein aggregates play a biphasic role, being a corollary of toxic mutant protein production early in disease process, and thus reflecting disease severity at early stages, but serving to sequester and mitigate mutant protein toxicity later in disease, and thus be inversely correlated with disease endpoints reflecting neuronal pathology at these later stages.

#### 3.3.12 NIIs—Correlation with behavior

Regression analysis for mutant mice by themselves also showed that some aspects of motor performance were improved by a higher aggregate burden. For example, the percent of the striatum covered by aggregates at 6 months of age was positively associated with the length of progression segments in open field (*r* = .524), suggesting that increased aggregate load improved this sign of motor endurance in open field. Note that, overall, 6‐month Q175 mice showed a trend toward a deficit compared to WT for the length of progression segments in open field. Thus, although the mutation seemingly adversely affected this motor parameter, among Q175 mice a larger striatal aggregate load at this age was associated with a mitigation of this hypokinetic sign. At 12 months, regression analysis for the mutant mice showed that rotarod performance was negatively correlated with the aggregate burden (% coverage) for striatum (*r* = −.508), indicating that higher aggregate load in striatum was associated with poorer rotarod performance. Note that at 12 months of age the mutant mice actually performed better than the WT on our accelerating rotarod task. At 18 months, progression segment length in open field was inversely correlated for Q175 mice with aggregate burden in Layer 5 of cerebral cortex (*r* = −.580), meaning greater aggregate abundance in presumptive corticostriatal neurons was associated with poorer motor performance, as reflected in shorter progression segment length. Moreover, at 18 months the increase in turn rate seen in Q175 mice (a possible sign of hyperkinesia) was highly correlated for Q175 mice with aggregate burden in Layers 5–6 of cerebral cortex (*r* = .637). Thus, disease magnitude (as reflected in aggregate load) in corticostriatal neurons was positively associated with this putative hyperkinetic behavior. Nonetheless, cortical aggregate abundance for Layers 2/3 and 5/6 combined was associated with a protective effect for rotarod at 18 months, since regression analysis for mutant mice showed that rotarod test performance was significantly positively correlated with the aggregate burden (% coverage) for cortical Layers 2, 3, 5, and 6 (*r* = .708). Our results thus suggest a complex role of mutant protein aggregates in pathogenesis and pathophysiology.

## 4 DISCUSSION

Given the use of the Q175 knock‐in HD mouse as a lead model for testing HD therapeutics, we sought to characterize the progression of striatal projection neuron, striatal interneuron, and striatal projection target pathology in 2‐, 6‐, 12‐, and 18‐month‐old male heterozygous Q175 mice. To aid in assessing the contribution of specific neuropathologies and neurochemical abnormalities to HD pathophysiology, we tested all mice on accelerating rotarod and open field behavior prior to being sacrificed for morphological–neurochemical analysis of pathology. In our neuropathological assessment, we focused on dSPN, iSPN, interneuron and overall neuron abundance in striatum, SPN terminal abundance in GPe, GPi, and SN, prototypical and arkypallidal neuron abundance in GPe, parvalbuminergic neuron abundance in GPi and SNr, dopaminergic neuron abundance in SNc, STN neuron abundance, upregulation of GABAA receptors in GPe, GPi, and SNr, and mutant protein aggregate load. Table 4 summarizes the major abnormalities seen in male heterozygous Q175 basal ganglia, most of which are already present at 6 months of age. In general, our findings show that neurochemical pathologies and behavioral abnormalities develop between 2 and 6 months of age in male heterozygous Q175 mice, and the overall level of neuropathology achieved by 18 months of age is less severe than that seen in humans at a premanifest stage of HD. Since prior studies have found no major difference between males and females in the Q175 phenotype (Goodliffe et al., 2018; Menalled et al., 2012; Padovan‐Neto et al., 2019; Rothe et al., 2015), it is likely this is true of female Q175 heterozygotes as well.

**TABLE 4:** Overall tally of major behavioral, morphological, and neurochemical endpoints examined in Q175 male heterozygous mice compared to WT mice at 2, 6, 12, and 18 months

Endpoint	2 months	6 months	12 months	18 months
*Behavior*	*Q175 as % WT*	*Q175 as % WT*	*Q175 as % WT*	*Q175 as % WT*
Weight	*−2.6%*	*‐3.6%*	**−24.1%**	**−18.5%**
Rotarod	0.4%	−4.9%	**13.1%**	**−13.7%**
Turn rate	−3.3%	**10.9%**	**19.9%**	**15.7%**
Center occupancy	0.3%	**−11.6%**	**−12.7%**	**−27.0%**
Max speed	2.9%	*−10.6%*	0.2%	**−10.9%**
Distance	3.1%	**−12.4%**	−1.3%	−2.6%
Progression segment length	12.5%	*−30.2%*	12.9%	−15.5%
*Striatal Perikarya counts*	*Q175 as % WT*	*Q175 as % WT*	*Q175 as % WT*	*Q175 as % WT*
Cresyl violet counts	Not done	0.6%	0.9%	−0.1%
NeuN counts	−0.9%	−1.2%	−4.5%	−0.1%
*Striatal volume*				
Fixed tissue	3.0%	−2.5%	**−4.9%**	**−4.9%**
Unfixed tissue	0.2%	**−11.1%**	**−11.2%**	**−14.4%**
*Striatal projection neuron counts*	*Q175 as % WT*	*Q175 as % WT*	*Q175 as % WT*	*Q175 as % WT*
ENK	−0.3%	**−15.3%**	**−15.8%**	**−25.5%**
SP	−2.0%	−5.0%	−0.1%	−7.0%
DARPP32	9.3%	**−22.9%**	**−20.6%**	**−43.3%**
*Striatal interneurons*	*Q175 as % WT*	*Q175 as % WT*	*Q175 as % WT*	*Q175 as % WT*
ChAT Perikarya count	Not done	10.7%	0.8%	2.7%
ChAT dendrite intersections	9.8%	**−21.9%**	**−24.2%**	Not done
ChAT dendrite volume	4.5%	**−20.6%**	**−21.0%**	Not done
PARV Perikarya counts	Not done	−14.6%	−10.3%	4.3%
nNOS Perikarya counts	Not done	Not done	Not done	5.8%
*Striatal projections*	*Q175 as % WT*	*Q175 as % WT*	*Q175 as % WT*	*Q175 as % WT*
ENK Striato‐GPe	−0.3%	**16.4%**	**10.1%**	**10.9%**
DARPP32 Striato‐GPe	−0.6%	4.6%	−8.1%	**−14.6%**
SP Striato‐GPi	3.2%	**36.4%**	**15.1%**	**23.8%**
DARPP32 Striato‐GPi	−2.7%	−5.2%	−3.0%	−15.4%
SP Striato‐SNr	3.9%	**14.0%**	**19.9%**	**9.4%**
DARPP32 Striato‐SNr	−6.4%	6.2%	−8.5%	4.9%
*Striatal neuronal targets*	*Q175 as % WT*	*Q175 as % WT*	*Q175 as % WT*	*Q175 as % WT*
GPe PARV prototypical count	Not done	−4.7%	4.6%	−4.4%
GPe FoxP2 Arkypallidal count	Not done	1.6%	−6.6%	**−22.0%**
GPe GABAA‐a1 ISHH intensity	−2.8%	0.6%	23.1%	7.9%
GPi PARV count	Not done	−2.1%	1.3%	11.3%
GPi GABAA‐a1 ISHH intensity	−7.3%	−1.4%	**41.6%**	17.3%
SNr PARV count	Not done	2.2%	−9.5%	3.5%
SNr GABAA‐a1 ISHH intensity	−3.0%	−1.6%	*28.7%*	5.1%
*Other basal ganglia structures*	*Q175 as % WT*	*Q175 as % WT*	*Q175 as % WT*	*Q175 as % WT*
STN neuron count	Not done	0.3%	−8.6%	**−25.9%**
SNc dopaminergic neuron count	Not done	−12.6%	−3.4%	−10.4%
*Mutant protein aggregates*	*Q175*	*Q175*	*Q175*	*Q175*
Cortex aggregate size in μm	0.0	0.0	2.02	2.09
Cortex aggregate % areal coverage	0.0%	0.0%	0.51%	1.36%
Striatum aggregate size in μm	0.0	1.56	2.03	1.85
Striatum aggregate % areal coverage	0.0%	0.33%	0.84%	1.89%

*Note*: In each case, the magnitude of the difference between mutant and WT is shown, with significant reductions or increases from WT shown in bold ‐. Trending differences are shown in italicized font.

In brief, weight shows WT—Q175 differential that increased from 2 to 12 months and then declined slightly by 18 months, with weight already trending toward significantly less in Q175 at 2 and 6 months of age. Although a rotarod deficit in Q175 mice was not present until 18 months of age, increased open field turn rate (reflecting hyperkinesia) and open field anxiety were a constant feature in Q175 from 6 months to 18 months. Somewhat oddly, Q175 mice performed better than WT mice on rotarod at 12 months of age. This may reflect the much lighter body weight of the Q175 mice, since we have found in our rotarod testing of mice that all other things being equal (or nearly so), lighter mice stay on the accelerating rotarod longer. For striatal perikarya, there was no overall significant loss of striatal neurons out to 18 months, but there was a deficiency in detectible ENK+ and DARPP32+ striatal perikarya (due apparently to reduced expression) already at 6 months that is more severe by 18 months. In contrast, there was no significant change in the abundance of detectible SP+ striatal perikarya over the 2‐ to 18‐month period. Moreover, striatal cholinergic, PARV, and NOS+ interneurons did not change in their abundance either, although cholinergic neurons showed significant dendrite attenuation in Q175 mice by 6 months of age. Despite the reduced ENK expression in striatal perikarya of the indirect pathway, the abundance of ENK‐immunostained terminals in GPe was significantly elevated at 6 months of age, and remained so out to 18 months of age. Similarly, the abundance of SP‐immunostained terminals from SP+ striatal direct pathway neurons in GPi and SN was elevated at 6 months of age, and also remained so out to 18 months. Although we saw no loss of prototypical PARV+ GPe pallidal neurons out to 18 months of age, there was a 20–30% loss of arkypallidal neurons as detected by FoxP2 immunolabeling at 18 months of age, and a 6.6% reduction at 12 months that trended toward significance. The presence of behavioral and SPN abnormalities as early as 6 months of age in Q175 male heterozygotes, which persist or grow worse, but not in 2‐month‐old Q175 male heterozygous mice, indicates that the mutation drives pathological progression after 2 months of age. These various findings are discussed below in more detail in relationship to findings in human HD and in prior mouse models of HD, notably prior studies on Q175 mice.

### 4.1 Striatal volume and total striatal neurons—Comparison to prior human and mouse studies

Neuropathological and imaging studies show that brain abnormalities in HD develop before evident symptoms, are progressive, and eventually involve the entire brain, resulting in about 25% brain weight loss in advanced HD (Halliday et al., 1998; Lange, 1981; Lange & Aulich, 1986; Lange et al., 1984; Sharp & Ross, 1996). Nonetheless, the most prominent neuropathology in HD occurs within the striatal part of the basal ganglia, in which gross atrophy is accompanied by extensive neuronal loss and astrogliosis, both of which become more severe as the disease progresses, with the atrophy leading to great enlargement of the lateral ventricles. Caudate shrinkage is significant already 10 years from estimated disease onset, while putamen and globus pallidus shrinkage is not significant until 3 years before estimated disease onset (Aylward et al., 1996, 2011; Tang et al., 2019; van den Bogaard et al., 2011). Consistent with these observations, we observed striatal shrinkage of about 10% relative to WT mice as early as 6 months in male heterozygous Q175 mice, which increased to about 15% by 18 months of age. Our findings are consistent with prior findings on heterozygous Q175 mice (Bertoglio et al., 2018; Heikkinen et al., 2012), which also reported a significantly smaller striatum by 6 months of age. Striatal atrophy is also commonly seen in other mutant mouse models of HD, such as R6/2 transgenic mice (Aggarwal et al., 2012; Mangiarini et al., 1996; Reiner et al., 2007; Reiner, Lafferty, et al., 2012; Sawiak, Wood, Williams, Morton, & Carpenter, 2009; Stack et al., 2005), YAC128 transgenic mice (Carroll et al., 2011), Detloff Q150 knock‐in mice (Heng, Tallaksen‐Greene, et al., 2008), and N171‐82Q transgenic mice (Aggarwal et al., 2012; Cheng et al., 2011), with the degree of atrophy depending on the state of progression in that particular model, and magnetic resonance imaging (MRI) more sensitive for detecting early changes. Notably, in male Q175 heterozygotes, striatal shrinkage is not accompanied by striatal neuron loss even long after striatal atrophy is first evident, although such loss is seemingly a concomitant of histologically evident striatal shrinkage in other HD models, such as R6/2 mice (Reiner, Lafferty, et al., 2012; Stack et al., 2005), YAC128 mice (Slow et al., 2003), Detloff Q150 knock‐in mice (Heng, Detloff, & Albin, 2008), and N171‐82Q mice (McBride et al., 2006; Ramaswamy et al., 2009). It may be that the striatal shrinkage we observed in Q175 mice in the absence of neuron loss stems from neuropil loss due to dendrite attenuation, as seen for cholinergic neurons by us and for PARV neurons by Holley et al. (2018). Goodliffe et al. (2018), however, reported no major attenuation of SPN dendrites in heterozygous Q175 mice, although Sebastianutto et al. (2017) reported attenuation of at least iSPN dendrites. Thus, it may be that the lower striatal water content in the mutant mice implied by the lesser volume difference between mutant and WT in the fixed than the fresh frozen contributes substantially to the lesser striatal volume of the mutants. This relative dehydration is likely to alter the brain milieu, and have pathophysiological implications (Pross, 2017; Tang et al., 2011; Vashisht, Morykwas, Hegde, Argenta, & McGee, 2018). The observation of increased thirst in human HD victims and in R6/2 mice (Wood et al., 2008) is consistent with the possibility that brain dehydration also occurs in human HD and other mouse HD models.

### 4.2 Striatal projection neurons—Functional implications and comparison to prior human and mouse studies

Based on markers of striatal terminals and in situ hybridization for SPNs, it is widely accepted that iSPNs are depleted earlier than dSPNs in human HD (Albin, Qin, Young, Penney, & Chesselet, 1991; Augood, Faull, & Emson, 1997; Augood, Faull, Love, & Emson, 1996; Deng et al., 2004; Glass et al., 2000; Reiner et al., 1988; Richfield et al., 1991; Richfield, Maguire‐Zeiss, Cox, et al., 1995; Richfield, Maguire‐Zeiss, Vonkeman, & Voorn, 1995; Richfield & Herkenham, 1994; Sapp et al., 1995). In the case of human HD, this appears to represent true loss of iSPNs (Albin et al., 1991; Richfield, Maguire‐Zeiss, Cox, et al., 1995; Richfield, Maguire‐Zeiss, Vonkeman, & Voorn, 1995). In the present study, we also observed that iSPNs are affected before dSPNs are affected in male Q175 heterozygous mice, as reflected in the preferentially diminished ENK expression in iSPNs, as well as the apparent preferential reduction in DARPP32 in them. Note that prior studies have also reported reduction in DARPP32 levels and DARPP32+ SPNs in striatum of heterozygous Q175 mice by 10–16 months of age, but not reported on whether it was differential between SPN types (Menalled et al., 2012; Smith et al., 2014; Rothe et al., 2015). Consistent with our findings, Menalled et al. (2012) reported reduced striatal expression of D2 dopamine receptors, an iSPN marker, by 4 months of age in male Q175 heterozygotes. Unlike in human HD, however, no actual loss of iSPNs was evident in our study out to 18 months of age, since our neuron counts detected no significant overall striatal neuron loss. Thus, in male Q175 heterozygous mice, the preferential iSPN pathology manifests as reduced expression of ENK and DARPP32, at least out to 18 months of age. Whether preferential iSPN loss occurs subsequently is not known, but seems likely since an overall 15% striatal neuron loss occurs by 10 months of age in homozygous Q175 mice, in which the phenotype is more aggressive (Heikkinen et al., 2012). Preferentially reduced expression of iSPN markers (such as ENK or D2 receptors) relative to dSPN markers (such as SP or D1 receptors) has also been reported in R6/2 mice (Cha et al., 1998; Luthi‐Carter et al., 2000; Menalled et al., 2000; Reiner, Lafferty, et al., 2012; Reiner, Wang, et al., 2012; Sun et al., 2002), YAC128 mice (Pouladi et al., 2012), and Q140 heterozygous mice (Rising et al., 2011). Menalled et al. (2014), however, observed similar loss for D1 and D2 expression in Q175 heterozygotes at 12 months of age, while we continued to observe greater loss of ENK than SP expression at this age, suggesting that perhaps D1 expression is more sensitive for detecting abnormalities in dSPNs, which do eventually develop during the course of HD progression in humans and presumably also in transgenic and knock‐in mouse models of HD.

The preferential loss of ENK+ iSPNs and ENK expression relative to SP+ dSPNs and SP expression in human HD is associated with and evidenced by depletion of ENK+ immunostaining from GPe, as early as pre‐manifest HD (Albin et al., 1990, 1992; Allen, Waldvogel, Glass, & Faull, 2009; Deng et al., 2004; Hedreen & Folstein, 1995; Sapp et al., 1995; Reiner, Dragatsis, & Dietrich, 2011). By Grade 4 of HD, however, profound loss in all projection systems is apparent (Albin, Reiner, Anderson, Penney, & Young, 1990; Reiner et al., 1988), with striato‐GPe and striato‐GPi projections at about 5% of normal (Deng et al., 2004). Diffusion tensor imaging (DTI) confirms massive loss of striatal projections in HD, indicating the immunolabeling changes reflect real fiber loss and not just staining loss (Douaud et al., 2009). Given that we also observed ENK expression loss by 6 months of age in male Q175 heterozygotes, it might be expected that, like in humans, this should be associated with reduction in ENK+ iSPN fibers and terminals in GPe. Instead, we observed an increase in ENK fibers and terminals in GPe beginning at 6 months that was still evident at 18 months of age. Moreover, although dSPNs were normal in abundance and SP expression, the abundance of SP fibers and terminals in GPi and SNr were also elevated. In contrast, ENK+ fibers in GPe are diminished in symptomatic R6/2 mice (Reiner, Lafferty, et al., 2012; Sun et al., 2002), consistent with their much more greatly reduced striatal ENK expression than in Q175 mice and their apparent loss of iSPNs. On the other hand, however, prior studies in R6/2 mice have found substantially elevated SP‐immunoreactive fiber abundance in nigra (Menalled et al., 2000; Reiner, Lafferty, et al., 2012; Sun et al., 2002), despite the only slightly reduced striatal expression of SP. Thus, both Q175 mice and R6/2 mice at symptomatic stages show the seemingly peculiar phenomenon that for SPN types showing only slightly reduced perikaryal neuropeptide expression but no or little neuron loss, terminal abundance for those neuropeptides in their target areas is increased. It may be that the increase in ENK terminals in GPe in Q175 mice, and SP terminals in GPi in Q175 mice and SP terminals in SN in both Q175 and R6/2 mice reflects a failure of neuropeptide release due to SPN dysfunction. Consistent with this interpretation, diminishing SPN activation via ablation of cortex results in increased SP levels in rat SN (Bouras, Vallet, & Hof, 1991; Somers & Beckstead, 1990), Q140 mice (from which Q175 are derived) have reduced cortical input to striatum (Deng, Wong, Bricker‐Anthony, Deng, & Reiner, 2013; Deng, Wong, Wan, & Reiner, 2014), and SPNs in awake, behaving R6/2 mice fail to show the burst activity that ensues from cortical activation (Miller, Walker, Shah, Barton, & Rebec, 2008; Rebec, Barton, & Ennis, 2002). Additionally, Barry, Akopian, Cepeda, and Levine (2018) showed reduced GABA release in SNr by dSPNs in R6/2 mice, and they attributed this effect to reduced activity of dSPNs. Similarly, in heterozygous Q175 mice at 6 months of age, Bevan and coworkers have shown that iSPNs show decreased responses to cortical activation (Callahan & Bevan, 2019). Thus, the presently observed increase in ENK+ striato‐GPe terminals and SP+ striato‐GPi/SN terminals in Q175 mice may, at least in part, reflect reduced neuronal firing of SPNs, and as a result, reduced neurotransmitter release by their terminals in GPe and GPi/SNr. Consistent with this possibility, we observed upregulation of GABAA receptors in GPi in our older Q175 mice. On the other hand, the increase in SPN terminals in GPe, GPi, and SN may represent a compensation for a deficiency in SPN inhibitory influence on output neurons of the striatal target areas. In particular, the sodium channel beta‐4 subunit (Scn4b) is one of the first genes downregulated in SPNs in Q175 mice and in HD (CHDI Foundation Provided Disclosure Notice 1; Oyama et al., 2006), is strongly responsive to CAG repeat length (CHDI personal communication), and is a prominent node in striatal transcriptional networks driven by repeat length identified in studies of an allelic series of HD knock‐in mice (CHDI personal communication). Moreover, the protein coded by Scn4b is highly abundant in SPNs (Miyazaki et al., 2014), is responsible for the resurgent current of sodium channels during polarization to negative potentials (Lewis & Raman, 2014), and thereby plays a critical role in the repetitive rapid firing of SPNs. Thus, the exuberant SPN innervation of its targets that we see already at 6 months of age in heterozygous male Q175 mice may represent a compensation for the dysregulation in SPN firing and resultingly reduced SPN inhibition of their target neurons caused by their early Scn4b reduction. In either case, it may be that Q175 mice reveal an early stage of SPN innervation of their target areas featuring terminal exuberance—one that occurs well before the typical HD patient death and neuropathological examination. It is also possible, on the other hand, that this phenomenon is characteristic of mouse HD models at stages prior to extensive SPN loss, and does not occur at any early stage of disease in human HD victims.

In addition to abnormalities in their expression patterns and their terminals in their target areas, SPNs in Q175 mice are also abnormal in their electrophysiology, for example showing increased membrane resistance and excitability by at least 8 months of age in heterozygotes (Goodliffe et al., 2018; Heikkinen et al., 2012; Indersmitten, Tran, Cepeda, & Levine, 2015; Sebastianutto et al., 2017). Both SPN types also appear to experience reduced cortical drive (Beaumont et al., 2016; Goodliffe et al., 2018; Heikkinen et al., 2012; Rothe et al., 2015), which our studies in Q140 mice suggest is likely to stem at least in part from reduced cortical input to striatum (Deng et al., 2013, 2014). Abnormalities in SPN function and their communication with their downstream target areas would be expected to affect motor functions, as occurs in human HD and as expected from the standard models of basal ganglia function (Albin et al., 1989; DeLong, 1990; Reiner, Dragatsis, & Dietrich, 2011). Prior studies of heterozygous Q175 mice have reported some hyperkinetic signs in the form of increased rearing at 4 months (Heikkinen et al., 2012) and increased stepping at 12 months (Smith et al., 2014). In our own study, we observed an increase in turn rate in open field, which we interpret as a hyperkinetic behavior. Regression analysis showed that the abundance of ENK‐ISHH perikarya in striatum was significantly inversely correlated with turn rate, meaning the reduction in ENK neurons in Q175 mice was associated with increased turning. Moreover, the abundance of DARPP32+ perikarya in striatum across the WT and Q175 cases at 6, 12, and 18 months of age was significantly inversely correlated with turn rate, meaning the reduction in DARPP32+ neurons in Q175 mice, which appears to preferentially involve iSPNs, was also associated with increased turning. At 18 months, both the loss of striatal DARPP32+ neurons and the loss of DARPP32+ fibers in GPe were significantly inversely correlated with the large increase in turn rate at that age. The linking of iSPN dysfunction, as reflected in reductions in both ENK and apparently DARPP32, to increased turning is of interest, as hyperkinesia is predicted for iSPN loss/hypofunction in the indirect–direct pathway model of basal ganglia function (Albin et al., 1989; DeLong, 1990; Reiner et al., 1988). Caution needs to be taken, however, in any inference made from these particular correlations, and any others we discuss, since the correlations could stem from a common impact of some other variable on the two correlated endpoints.

The reduction in iSPNs was, however, also associated with hypokinetic signs such as lessened distance traveled and slowing. For example, the abundance of ENK‐ISHH perikarya in striatum was significantly correlated with distance traveled in open field and the length of progression segments in open field, meaning fewer ENK perikarya led to less distance traveled in open field and shorter units of progression. At 6 months of age, when speed was reduced in mutant mice, the abundance of ENK‐ISHH perikarya was highly and significantly correlated with maximum speed in open field, suggesting the lessening of ENK‐labeled neurons in Q175 mice may have led to slowing. Similarly, striatal DARPP32 neuron abundance across the WT and Q175 cases at 6, 12, and 18 months of age was significantly correlated with distance traveled in open field and maximum speed in open field, meaning fewer DARPP32+ perikarya may have led to less distance traveled in open field and slower progression. At 18 months, when a rotarod deficit was seen, regression analysis showed that striato‐GPe ENK fiber abundance was significantly inversely correlated with rotarod performance. It may be that the hyperkinetic movements ensuing from iSPN dysfunction also impair overall motor performance, causing reduced distance and speed in open field and reduced time on rotarod.

### 4.3 Striatal interneurons—Functional implications and comparison to prior human and mouse studies

Three major types of striatal interneurons have been identified: (a) tonically active aspiny cholinergic neurons (TANs; Bennett, Callaway, & Wilson, 2000; Kawaguchi, Wilson, Augood, & Emson, 1995); (b) low threshold spiking (LTS) aspiny neurons that contain GABA, somatostatin (SS), neuropeptide Y (NPY), and nNOS (Figueredo‐Cardenas, Morello, Sancesario, Bernardi, & Reiner, 1996; Kawaguchi et al., 1995); and (c) aspiny fast‐spiking interneurons (FSIs) that contain GABA and PARV (Kawaguchi et al., 1995; Kita, Kosaka, & Heizmann, 1990). Cholinergic and SS+ striatal interneurons are resistant in HD, and survive even late into the disease (Albin, Reiner, et al., 1990; Cicchetti, Prensa, Wu, & Parent, 2000; Dawbarn, DeQuidt, & Emson, 1985; Ferrante, Beal, et al., 1987; Ferrante et al., 1985, 1986; Ferrante, Kowall, et al., 1987; Hawker & Lang, 1990; Kowall, Ferrante, & Martin, 1987; Massouh, Wallman, Pourcher, & Parent, 2008; Norris, Waldvogel, Faull, Love, & Emson, 1996; Richfield, Maguire‐Zeiss, Cox, et al., 1995; Sapp et al., 1995). The preservation of cholinergic interneurons in HD striatum is nonetheless accompanied by diminished expression of such cholinergic neuron markers as ChAT (Aquilonius, Eckernas, & Sundwall, 1975; Ferrante, Beal, et al., 1987; Massouh et al., 2008; Spokes, 1980; Suzuki, Desmond, Albin, & Frey, 2001) and the vesicular acetylcholine transporter (VAChT; Smith et al., 2006). Similarly, although SS+ neuron abundance does not decline in HD striatum, expression of nNOS and SS in these neurons is progressively diminished (Norris et al., 1996). PARV+ interneurons are lost from the striatum as HD progresses at a rate somewhat slower than that of iSPNs and this loss may contribute to the dystonia that appears as disease moves past the early choreiform stage (Reiner et al., 2013).

Like in human HD, we observed no loss of cholinergic TANs, but we did note dendritic tree attenuation for cholinergic neurons beginning at 6 months of age, as we had previously for Q140 mice (Deng & Reiner, 2016), from which the Q175 mice are derived. In that prior study, we showed a 35% reduction in thalamic terminals on cholinergic interneuron dendrites in heterozygous Q140 striatum, largely due to the ChAT+ dendrite loss. Our findings of abnormalities in cholinergic striatal interneuron dendritic branching, but preservation of neuronal numbers, is consistent with prior studies in mouse models of HD. For example, Smith et al. (2006) reported that despite a normal number of cholinergic interneurons in the striatum in R6/1 HD transgenic mice, the levels of both the vesicular acetylcholine transporter (VAChT) and ChAT are markedly decreased. Similarly, studies of other HD models (Holley et al., 2015; Vetter et al., 2003) have reported preserved neuron numbers but depressed ChAT activity and VAChT binding. Farrar et al. (2011) noted that activation of excitatory inputs to striatal cholinergic interneurons is dysfunctional in R6/2 HD mice, and suggested that the reduced levels of extracellular striatal ACh in HD striatum may reflect abnormalities in the excitatory innervation of cholinergic interneurons, while Holley et al. (2015) reported enhanced inhibitory responses in striatal cholinergic interneurons in R6/2 mice, which also could contribute to reduced acetylcholine release. Cholinergic interneurons represent 1–2% of all striatal neurons and receive their major excitatory input from the thalamus, as well as an input from midbrain dopaminergic neurons that exerts an inhibitory effect via D2 receptors on cholinergic interneurons (Lapper & Bolam, 1992; Pisani, Bernardi, Ding, & Surmeier, 2007). Cholinergic interneurons innervate both iSPNs and dSPNs, and modulate SPN responsivity and plasticity at corticostriatal synapses (Ding, Guzman, Peterson, Goldberg, & Surmeier, 2010; Pisani et al., 2007; Smith, Surmeier, Redgrave, & Kimura, 2011). Thalamic activation of cholinergic interneurons, in particular, is thought to play a role in attentional shifts to salient or novel environmental stimuli mediated by striatum, via the influence of cholinergic interneurons on SPN function. Abnormalities in thalamic input to striatal cholinergic interneurons may help explain the early hyperkinesia reported for Q140 mice, as the loss of thalamic input to cholinergic neurons would be predicted to more greatly lessen the responses of iSPNs than dSPNs to cortical drive (Smith et al., 2011), which models of basal ganglia function predict should cause hyperkinesia (Albin et al., 1989).

Also like in human HD, we observed no loss of nNOS+ LTS interneurons in Q175 male heterozygotes at 18 months of age. Because there was no loss at this age, we did not examine earlier ages. Perhaps because of their hardiness in human HD, LTS neurons have been little evaluated in mutant mouse HD models, and there are no reports of their abundance in other models. The striatal LTS interneurons co‐containing SS, NPY, and NOS are GABAergic, and makeup about 0.5–1% of all striatal neurons (Koos & Tepper, 1999; Tepper, Tecuapetla, Koós, & Ibáñez‐Sandoval, 2010). They receive a weak cortical input, and have a modulatory somatostatinergic and weak GABAergic influence on SPNs (Galarraga et al., 2007). By means of NO release acting on SPN cGMP signaling, LTS interneurons also facilitate SPN responses to cortical activation (Sammut, Park, & West, 2007; West & Grace, 2004). Although somatostatinergic neuron abundance does not decline in HD striatum, NOS, and SS expression is progressively diminished (Norris et al., 1996). Moreover, LTS interneurons in at least some mouse HD models (R6/2 and BACHD) have been shown to be more spontaneously active than normal (R6/2 and BACHD; Cepeda et al., 2013). Nonetheless, SPN responses to activation of LTS interneurons were normal in these same mice. In our own study of Q175 mice, the morphology of LTS neurons did not obviously differ from age‐matched WT. It is thus not evident that any major abnormalities exist for this neuron type in mouse models of HD, or that any contribute notably to the HD phenotype in humans.

Unlike in human HD, we observed no loss of PARV+ FSIs. Although we did not assess dendritic branching of PARV interneurons in the Q140 mice, Holley, Galvan, Kamdjou, Cepeda, and Levine (2018) have reported that they are attenuated. Loss of striatal FSIs has been reported in some but not all studies in HD mice. For example, Paldino et al. (2017) reported about a 30% loss in 13‐week‐old highly symptomatic R6/2 mice associated with dendrite loss for surviving PARV neurons, while Cabanas et al. (2017) reported reduced activation of striatal FSIs in R6/1 mice. In contrast, Simmons et al. (2013) reported no PARV interneuron loss in 10‐week‐old symptomatic R6/2 mice, but considerable neuronal shrinkage and dendrite attenuation. Given that PARV+ striatal interneuron loss in human HD becomes prominent subsequent to the early wave of iSPN loss, it seems likely that PARV+ striatal interneurons are not lost in mouse HD models until after iSPN loss occurs, as for example in late symptomatic R6/2 mice (Reiner, Lafferty, et al., 2012; Sun et al., 2002). PARV+ striatal interneurons, which use GABA as their neurotransmitter, represent only 1–2% of striatal neurons (Deng, Shelby, & Reiner, 2010; Kawaguchi et al., 1995; Kita, 1993), and are about twice as abundant in lateral motor striatum as medial non‐motor striatum (Deng et al., 2010). PARV+ striatal interneurons receive cortical input, have high basal firing rates, and have a powerful feedforward inhibitory input to the perikarya and dendrites of nearby SPNs (Koos & Tepper, 1999; Lapper, Smith, Sadikot, Parent, & Bolam, 1992; Rudkin & Sadikot, 1999; Tepper, Koos, & Wilson, 2004), apparently preferentially to dSPNs (Gittis, Nelson, Thwin, Palop, & Kreitzer, 2010; Planert, Szydlowski, Hjorth, Grillner, & Silberberg, 2010). Developmental delay in the maturation of these neurons results in an intermittent subtype of dystonia, called paroxysmal dystonia, in a spontaneously arisen mutant Syrian hamster (the *dt* ^*sz*^ hamster; Bennay, Gernert, & Richter, 2001; Gernert, Bennay, Fedrowitz, Rehders, & Richter, 2002; Gernert, Hamann, Bennay, Löscher, & Richter, 2000; Gernert, Richter, & Löscher, 1999a, 1999b, 1999c). The diminished feedforward inhibition is associated with increased firing of dSPNs and decreased firing of GPi neurons, and the resulting increased firing of motor thalamus may yield inappropriate activation of antagonistic muscle groups, and be the basis of the dystonia seen in the *dt* ^*sz*^ hamsters. Consistent with a role of PARV+ striatal interneuron deficiency in at least some forms of dystonia, Gittis et al. (2011) showed that infusion of IEM‐1460 (an inhibitor of the GluA2‐lacking AMPA receptors characteristic of FSIs) into mouse sensorimotor striatum, which selectively blocks synaptic excitation of PARV interneurons, elicits dystonia‐like movements. Moreover, haplo‐insufficiency of *Nkx2.1* in humans, likely leading to subnormal colonization of striatum by PARV+ interneurons, causes dystonia (Breedveld et al., 2002) and recording studies in humans show that, like in *dt* ^*sz*^ hamsters, GPi neuronal firing is reduced in dystonic individuals (Hashimoto, 2000; Hutchison, Lang, Dostrovsky, & Lozano, 2003; Sanghera et al., 2003; Tang et al., 2007; Vitek et al., 1999; Zhuang, Li, & Hallett, 2004). Thus, dysfunction or loss of this neuron type may contribute to the HD symptom of dystonia. Increased responsiveness of SPNs to the FSI input may compensate for their decline early in the course of HD in humans and mouse models (Cepeda et al., 2013).

### 4.4 Striatal target neurons—Functional implications and comparison to prior human and mouse studies of GPe, GPi, and SNr

The GPe in rodents has been shown to contain two major populations of output neurons: (a) prototypical GABAergic pallidal neurons that are more laterally situated, express PARV, make up about 55% of all GPe neurons, and project to STN and parafascicular nucleus of the thalamus; and (b) arkypallidal GABAergic neurons that are more medially situated, express FoxP2 but not PARV, make up about 25% of all GPe neurons, and project back to SPNs of dorsolateral motor striatum (Abdi et al., 2015; Dodson et al., 2015; Gittis et al., 2014; Glajch et al., 2016; Hegeman et al., 2016; Hernández et al., 2015; Mallet et al., 2012; Mastro et al., 2014; Sato et al., 2000). In contrast, GPi and SNr predominantly contain PARV+ pallidal‐type projection neurons, although some calretinin‐containing neurons are present (Lee & Tepper, 2007). We found that the GPe, GPi and SNr in Q175 mice were largely normal in their prototypical PARV+ pallidal type GABAergic neurons, as determined from immunolabeling and from ISHH analysis from 6 to 18 months of age. Arkypallidal FoxP2+ GPe neurons, however, showed loss at 18 months, which seemed to be already in its early stages at 12 months. As arkypallidal neurons project back to SPNs of dorsolateral motor striatum (Abdi et al., 2015; Hernández et al., 2015) and their activation influences motor function (Glajch et al., 2016), their loss at 18 months would have an impact on basal ganglia motor function. Prior studies in other mouse HD models have not examined survival of GPe, GPi, or SNr neurons, but Perez‐Rosello et al. (2019) reported that Q175 mice show increased GPe responsiveness to striatal input at 6 months of age, as well as in symptomatic 9‐week‐old R6/2 mice, in large part due to increased postsynaptic response efficacy. This enhanced responsiveness increased the ability of striato‐GPe axon terminals to inhibit ongoing activity in prototypical neurons. The basis of the increased efficacy was unclear, but our current data suggests it is not due to an increase in GABAA receptors on GPe neurons. In any case, this adaptation could help compensate for a deficit in the ability of iSPNs to pause prototypical GPe neuron activity due to the loss of iSPNs or a diminished excitability of iSPNs by their cortical input, which would otherwise promote hyperkinesia. Note, however, that the phenomenon of increased responsiveness of GPe neurons to iSPN activation was not seen in YAC128, R6/1, or R6/2 mice (Akopian, Barry, Cepeda, & Levine, 2016; Barry et al., 2018; Du et al., 2016), and may represent an early stage of HD pathophysiology prior to SPN loss.

Arkypallidal neurons are thought to mediate a stop signal to SPNs (Mallet et al., 2016), and so their loss at 18 months in Q175 mice might contribute to the increased hyperkinesia seen at this age. In HD, significant progressive atrophy occurs in GPe and GPi, with greater atrophy and gliosis in GPe than GPi (Douaud et al., 2006; Halliday et al., 1998; Lange, 1981; Roos, 1986; Vonsattel, 2008; Vonsattel & DiFiglia, 1998). The atrophy and gliosis are evident by Grade 3, and prominent by Grade 4 (50%). The shrinkage appears to be due to both neuron loss and loss of striatal input (Lange, Thörner, Hopf, & Schröder, 1976). Of note, pallidal shrinkage seems more diagnostic of symptom onset than does striatal shrinkage, since imaging studies show that striatal shrinkage occurs well before symptoms are manifest, but pallidal shrinkage more immediately precedes symptom appearance (Aylward et al., 1996, 2011; Tang et al., 2019; van den Bogaard et al., 2011). The pathophysiological contribution of these pallidal changes is uncertain. In principle, the preferential loss of GPe neurons seen in humans would disinhibit the subthalamic nucleus and contribute to akinesia and possibly rigidity, and could thus be a contributor to these late HD symptoms. SN pars reticulata undergoes cell loss and shrinkage in HD as well, but less so than does striatum (Byers, Gilles, & Fung, 1983; Cudkowicz & Kowall, 1990; De La Monte, Vonsattel, & Richardson Jr., 1988; Sharp & Ross, 1996; Vonsattel et al., 1985). Loss of SNr GABAergic neurons is evident in HD (Vonsattel, 2008), and Oyanagi, Takeda, Takahashi, Ohama, and Ikuta (1989) have reported 40% loss in late HD. Shrinkage of SN as detected by CT imaging also has been reported, which could stem from both striatal input loss and SNr neuron death (Douaud et al., 2006). Loss of GPi and SNr neurons would be expected to reduce inhibition of motor thalamus, causing excessive thalamic drive to motor cortex and disrupting initiation of voluntary behavior by the Go‐dSPNs of the direct pathway. It may be, however, that this effect is counterbalanced and overcome by the even greater loss of GPe neurons, or the loss of dSPNs.

### 4.5 SN pars compacta—Functional implications and comparison to prior human and mouse studies

The dopaminergic input to the striatum from the SNc heavily targets SPNs and cholinergic interneurons, and thereby plays a major role in motor plasticity and performance (Reiner & Deng, 2018). The importance of this role is evidenced by the motor impairments ensuing from its extensive loss in Parkinson's disease. Although we saw no loss of dopaminergic neurons in Q175 mice out to 18 months of age, Smith et al. (2014) reported reduced dopamine levels in Q175 heterozygotes at 12 and 16 months of age. Similarly, in R6/2 and R6/1 mice, progressive reduction in striatal DA release is seen beginning at 6 weeks of age (Cepeda, Murphy, Parent, & Levine, 2014; Mochel, Durant, Durr, & Schiffmann, 2011; Petersen et al., 2002), as well as reductions in striatal homovanillic acid in later symptomatic stages (Mochel et al., 2011). Loss of SNc dopaminergic neurons is eventually evident in HD (Vonsattel, 2008), with about 40% loss of neurons and dopaminergic neuron markers such as tyrosine hydroxylase reported late in HD (Oyanagi et al., 1989; Yohrling IV et al., 2003). Moreover, dopaminergic neurons have been reported to be 33% smaller than normal in human HD. As expected from loss of nigral dopaminergic neurons and reduced tyrosine hydroxylase expression, terminals containing tyrosine hydroxylase are reduced in their abundance in advanced HD striatum (Bedard et al., 2011; Ferrante & Kowall, 1987). Consistent with this, dopamine, homovanillic acid (HVA), the dopamine transporter (DAT), and the vesicular monoamine transporter‐2 (VMAT2) also have been reported to be reduced in HD striatum in late disease (Bohnen et al., 2000; Ginovart et al., 1997; Kish, Shannak, & Hornykiewicz, 1987; Suzuki et al., 2001). Nonetheless, increases in dopamine have actually been observed early in disease (Garrett & Soares‐da‐Silva, 1992). Late loss of dopamine input could contribute to akinesia in HD, as it does in Parkinson's disease, while early increases could synergize with iSPN loss and dysfunction to exacerbate hyperkinesia. In the latter regard, it is interesting to note that L‐DOPA provokes hyperkinesia in otherwise asymptomatic HD carriers (Klawans, Paulson, & Barbeau, 1970).

### 4.6 Subthalamic nucleus—Functional implications and comparison to prior human and mouse studies

The subthalamic nucleus is a key part of basal ganglia circuitry, being activated by a direct cortical excitatory input via the hyperdirect basal ganglia pathway and a disinhibitory input via the iSPN‐GPe link of the indirect pathway way (Albin et al., 1989; Oldenburg & Sabatini, 2015). As such, the STN provides an excitatory signal to the GPi that is thought to suppress potentially conflicting behaviors during action selection by the direct pathway. Consistent with this scenario, degeneration of iSPNs is associated with the choreiform symptoms of early HD and direct destruction of STN causes the hyperkinesia of ballismus (Albin et al., 1989). As did Atherton et al. (2016), we found significant STN neuron loss in older Q175 heterozygotes, in their case at 12 months, and in our case at 18 months. They also showed similar loss of STN neurons in BACHD mice. Notably, striatal neuron loss is absent in both mice at these ages at which STN neuron loss is significant (Gray et al., 2008; Heikkinen et al., 2012; Mantovani et al., 2016; Smith et al., 2014). Even prior to the STN neuron loss, however, diminished activity of STN neurons has been observed in Q175 and BACHD mice (Atherton et al., 2016). STN hypoactivity has also been observed in YAC128 and R6/2 mice (Callahan & Abercrombie, 2015a, 2015b). Such STN dysfunction could contribute to the early motor hyperactivity we saw in our Q175 mice. STN neuron loss appears to be a late event in human HD, with up to 25% volume and neuron loss by late HD (Guo et al., 2012; Lange, 1981; Lange & Aulich, 1986; Lange et al., 1976, 1984), although it is uncertain to what extent volume loss reflects neuron loss or loss of GPe and or cortical input. The studies of mutant mice suggest early dysfunction may precede the late STN loss and play a role in early choreic symptoms.

### 4.7 Mutant protein aggregates—Pathogenic implications and comparison to prior human and mouse studies

Mutant protein aggregates are a hallmark of HD brain in humans, yet their localization has not been consistently associated with disease progression. Neuropathological studies in humans suggest that the pathogenic HD gain of function could be the formation of ubiquitinated aggregates of the N‐terminal fragment of mutated huntingtin, which is thought to occur due to enhanced cleavage and aggregation of the polyglutamine rich part of the mutant huntingtin N‐terminus (DiFiglia et al., 1997; Gutekunst et al., 1999; Li & Li, 1998; Maat‐Schieman et al., 1999; Martindale et al., 1998; Sieradzan et al., 1999; Vonsattel, 2008). Aggregates of mutant protein are observed in neocortex, entorhinal cortex, subiculum, hippocampal pydamidal neurons, and striatum, more so in advanced and/or juvenile onset HD (Figure 3). Aggregates are, however, rare in globus pallidus, SN, and cerebellum in human HD. Formation of NIIs in HD brain, however, is not prominent in cerebral cortex until advanced stages of HD and is never prominent in striatum (1–4% of neurons) at any stage (DiFiglia et al., 1997; Gutekunst et al., 1999; Kuemmerle et al., 1999; Sapp et al., 1999). In fact, the striatal neurons that do possess NIIs tend to be interneurons, which survive well in HD, rather than projection neurons (Kuemmerle et al., 1999). This brings into question if NIIs are, in fact, pathogenic (Reiner, Dragatsis, Zeitlin, & Goldowitz, 2003). Neuropil aggregates (found in spines, dendrites, and axons) are far more common in HD cortex and striatum than NIIs, and thus may be pathogenic by interfering with neuronal function, particularly corticostriatal communication (DiFiglia et al., 1997; Gutekunst et al., 1999; Kuemmerle et al., 1999; Sapp et al., 1999). In juvenile‐onset HD patients (which mouse models tend to resemble due to their relatively extreme CAG repeat lengths compared to humans with adult onset), NIIs are more prevalent than cytoplasmic and neuropil aggregates, while in adult human HD brains the latter is more common (DiFiglia et al., 1997). Mutant mouse models also provide mixed evidence for the pathogenic role of mutant protein aggregates. For example, in R6/2 mice, the early accumulation of NIIs in cortex is consistent with the early appearance of motor and learning abnormalities in these mice, and the eventual pervasiveness of NIIs at ages at which severe abnormalities are evident is consistent with their contribution to a neuronal dysfunction underlying the abnormalities (Meade et al., 2002). That cortex develops larger NIIs than striatum, however, is inconsistent with a role of them in the preferential loss of striatal neurons in HD (Meade et al., 2002). In 12‐ to 18‐month‐old BACHD mice, neuropil aggregates predominate, and are far more common in cortex than striatum, as in adult‐onset human HD (Gray et al., 2008). In YAC128 mice, diffuse nuclear localization of mutant protein with small aggregates has been reported, rather than the large NIIs seen in R6/2 mice, and this appears first in striatal neurons in YAC128 mice (Slow et al., 2003; Van Raamsdonk, Murphy, Slow, Leavitt, & Hayden, 2005). This is also the case in the Detloff Q150 mice (Lin et al., 2001; Tallaksen‐Greene, Crouse, Hunter, Detloff, & Albin, 2005). We observed that in Q175 heterozygous male mice aggregates formed in striatal neurons by 6 months, and cortical aggregates were not seen until our 12‐month‐old mice. All or nearly all striatal neurons contained aggregates. Similarly, in heterozygous Q175 mice, Carty et al. (2015) observed EM48‐positive mutant protein nuclear inclusions in striatum at 4 months, including within SPNs, while nuclear inclusions in cortex were undetectable until at least 8 months of age. As in our study, aggregates were seen to increase in size over time. During progression, we also observed an increase in neuropil aggregates in Q175 heterozygotes, as did Carty et al. (2015). Although the preferential appearance of mutant protein aggregates in striatum is consistent with a role for them in driving preferential striatal pathogenesis in HD, it still could be that they are protective and a byproduct of generation of smaller toxic mutant protein fragments. In this regard, our own data on the relationship of the aggregates to pathology and dysfunction are of interest.

In general, we found that a higher striatal aggregate load was associated with a lessening of striatal neuron or projection neuropathology. For example, among 6‐month‐old Q175 mice, the percent of the striatum covered by aggregates tended to inversely correlate with the abundance of ENK‐immunostained GPe fibers and the abundance of SP‐immunostained SN fibers, providing suggestive evidence that the abnormally increased abundance of ENK‐immunostained fibers in GPe and of SP‐immunostained fibers in SN was less great in Q175 mice with greater aggregate burden. A similar result for SP+ fiber abundance in GPi was seen in Q175 mice at 12 months. For DARPP32+ SPNs, the aggregate abundance was again associated with a protective effect, with striatal aggregate load strongly positively correlated with DARPP32+ neuron abundance among 12‐month‐old Q175 mice and 18‐month‐old mice. Increasing aggregate load in striatum was, however, not consistently correlated with a better motor outcome. For example, although the percent of the striatum covered by aggregates at 6 months of age was positively associated with the length of progression segments in open field, the striatal aggregate load was significantly inversely correlated with distance traveled in open field and highly inversely correlated with maximum speed. At 12 months, regression analysis for the mutant mice showed that rotarod performance was significantly negatively correlated with the aggregate burden for striatum, indicating that higher aggregate load in striatum was associated with poorer rotarod performance. At 18 months, a decrease in progression segment length in open field was significantly inversely correlated for Q175 mice with aggregate burden in Layers 5–6 of cerebral cortex, meaning greater aggregate abundance in presumptive corticostriatal neurons was associated with poorer motor performance on progression segment length. Similarly, at 18 months the increase in turn rate seen in Q175 mice (a possible sign of hyperkinesia) was highly correlated for Q175 mice with aggregate burden in Layers 5–6 of cerebral cortex, meaning increased deep cortical aggregate burden led to more hyperkinesia. Thus, disease magnitude (as reflected in aggregate load) in corticostriatal neurons was positively associated with behavioral abnormalities. Nonetheless, cortical aggregate abundance at 18 months was associated with a protective effect for rotarod, since regression analysis for mutant mice showed that rotarod performance was significantly positively correlated with the aggregate burden (% coverage) for cortical layers 2, 3, 5, and 6. Our results thus reflect a complex role of mutant protein aggregates in pathogenesis and pathophysiology, generally suggesting that they protect against pathogenesis but cause neuronal dysfunction that hinders behavior.

## CONFLICT OF INTEREST

None of the authors has any interest or relationship that might be perceived as influencing their objectivity.

## AUTHOR CONTRIBUTIONS

Yunping Deng conducted the analysis in the immunolabeling studies. Hongbing Wang conducted the analysis in the in situ studies. Marion Joni performed some of the analysis in the immunolabeling studies. Radhika Sekhri performed some of the analysis in the immunolabeling studies. Anton Reiner planned and supervised the studies, participated in analysis, and wrote the manuscript, and made its illustrations.

## ETHICS STATEMENT

All work was conducted in a manner consistent with Journal guidelines regarding ethical and responsible research conduct.

### PEER REVIEW

The peer review history for this article is available at https://publons.com/publon/10.1002/cne.25023.

## Data Availability

The data used and/or analyzed during the current study are available from the corresponding author on reasonable request.
